# The emerging role of Deubiquitinases (DUBs) in parasites: A foresight review

**DOI:** 10.3389/fcimb.2022.985178

**Published:** 2022-09-27

**Authors:** Prakash Kumar, Pawan Kumar, Debabrata Mandal, Ravichandiran Velayutham

**Affiliations:** ^1^ Department of Biotechnology, National Institute of Pharmaceutical Education and Research, Hajipur, India; ^2^ National Institute of Pharmaceutical Education and Research, Kolkata, India

**Keywords:** deubiquitination, parasitic disease, drug target, *in-silico* analysis, ubiquitin, USP, DUB domains

## Abstract

Before the discovery of the proteasome complex, the lysosomes with acidic proteases and caspases in apoptotic pathways were thought to be the only pathways for the degradation of damaged, unfolded, and aged proteins. However, the discovery of 26S and 20S proteasome complexes in eukaryotes and microbes, respectively, established that the degradation of most proteins is a highly regulated ATP-dependent pathway that is significantly conserved across each domain of life. The proteasome is part of the ubiquitin-proteasome system (UPS), where the covalent tagging of a small molecule called ubiquitin (Ub) on the proteins marks its proteasomal degradation. The type and chain length of ubiquitination further determine whether a protein is designated for further roles in multi-cellular processes like DNA repair, trafficking, signal transduction, etc., or whether it will be degraded by the proteasome to recycle the peptides and amino acids. Deubiquitination, on the contrary, is the removal of ubiquitin from its substrate molecule or the conversion of polyubiquitin chains into monoubiquitin as a precursor to ubiquitin. Therefore, deubiquitylating enzymes (DUBs) can maintain the dynamic state of cellular ubiquitination by releasing conjugated ubiquitin from proteins and controlling many cellular pathways that are essential for their survival. Many DUBs are well characterized in the human system with potential drug targets in different cancers. Although, proteasome complex and UPS of parasites, like plasmodium and leishmania, were recently coined as multi-stage drug targets the role of DUBs is completely unexplored even though structural domains and functions of many of these parasite DUBs are conserved having high similarity even with its eukaryotic counterpart. This review summarizes the identification & characterization of different parasite DUBs based on *in silico* and a few functional studies among different phylogenetic classes of parasites including Metazoan (*Schistosoma*, *Trichinella*), Apicomplexan protozoans (*Plasmodium*, *Toxoplasma*, *Eimeria*, *Cryptosporidium*), Kinetoplastidie (*Leishmania*, *Trypanosoma*) and Microsporidia (*Nosema*). The identification of different homologs of parasite DUBs with structurally similar domains with eukaryotes, and the role of these DUBs alone or in combination with the 20S proteosome complex in regulating the parasite survival/death is further elaborated. We propose that small molecules/inhibitors of human DUBs can be potential antiparasitic agents due to their significant structural conservation.

## Introduction

Scientists Joseph Etlinger and Alfred L. Goldberg showed, in the late 1970s, that protein degradation happens in reticulocytes, which lack lysosomes, suggesting the presence of a second intracellular degradation mechanism that is ATP-dependent (Etlinger and Goldberg, 1977). Later, the identification of unexpected covalent modification ubiquitination, of the histone protein by ubiquitin, which itself is a small peptide/protein of a length of ~76 amino acids, unravels the importance of enzymes involved in ubiquitination and deubiquitinations pathway (Goldknopf and Busch, 1977). The importance of proteolytic degradation inside cells and the role of ubiquitination/deubiquitination in proteolytic pathways was a key scientific discovery that leads to the 2004 Nobel Prize award in Chemistry to Dr. Aaron Ciechanover, Dr. Avram Hershko, and Dr. Irwin Rose. The detailed atomic structures in seven different conformational states at 2.8-3.6Å resolution of the human 26S proteasome holoenzyme in a complex with a polyubiquitylated protein substrate were solved by cryogenic electron microscopy (cryo-EM) in 2018 (Dong et al., 2019). These structures revealed the mechanisms by which the protein substrate is recognized after ubiquitination/deubiquitination, and degraded by the human 26S proteasome in a series of multistep coordinated processes. In the last two decades, extensive research has revealed that ubiquitination and deubiquitination machinery controls a wide range of cellular functions, such as degradation of damaged, aged & unfolded proteins, DNA repair, chromatin remodeling, cell cycle regulation, cell signaling pathways, etc. Further, it degrades protein to salvage the peptides and amino acids for re-utilization (Wilkinson, 1997). The generation of assembly between the C terminal of Glycine of target proteins and any of ubiquitin’s seven Lysine residues(Lys6, 11, 27, 29, 33, 48, and 63) by isopeptide bond formation leads to the formation of mono and poly-ubiquitin chains that diversifies ubiquitylation (Komander et al., 2009). Deubiquitinating proteases (DUBs) are a class of hydrolases that cleave the poly-ubiquitin chain to remove multiple ubiquitin residues into mono-ubiquitin. The length of ubiquitin chain after ubiquitination/deubiquitination on the proteins is a deciding factor where the protein will go for degradation in proteasome or trafficked to other parts of the cell to participate in many cellular pathways including signal transduction, DNA repair, cell cycle regulation, apoptosis, etc. Further, since the UPS and DUBs target their proteins based on their domain structure and recognition, the fate of the modified protein is determined by a complex equilibrium of conjugation by UPSs and deconjugation by DUBs (Wei et al., 2015).

Despite the sequencing of many parasite genomes, enzymes of the ubiquitination and deubiquitination pathways; which are conserved and, probably, important for their survival; have not been well studied to date in parasites. Several enzymes involved in the deubiquitination process in humans have been used as drug targets in different disease domains including cancer, viral diseases, and neurodegenerative disorders but the study is yet to provide a successful clinical application (Daviet and Colland, 2008; Colland, 2010; Nicholson et al., 2014; D'Arcy et al., 2015; Farshi et al., 2015; Ndubaku and Tsui, 2015; Gupta et al., 2018). However, the United States Food & Drug Administration (USFDA) approval of the anti-cancer drug bortezomib in 2003 (Velcade by Takeda Pharmaceuticals) which targets the mammalian proteasome complex (Kane et al., 2003) reveals the critical importance of UPS and DUBs as drug targets. The recent cryo-EM structure of *Plasmodium falciparum* 20S proteasome bound to the inhibitor at 3.6 Å resolution and (Li et al., 2016) *Leishmania tarentolae* proteasome in complex with LXE408 (Nagle et al., 2020), a small molecule with triazolopyrimidine scaffold, at 3.4 Å showed that the proteasome complex and the associated UPS/DUB can be potential drug targets in parasites also. The molecule LXE408 is currently in phase I clinical trial for the treatment of leishmaniasis. Even though more than 100 DUBs were characterized in humans, not a single DUB from parasite was characterized with detailed biochemical and functional studies. It is important to note that, DUBs target the proteins in a single-step reaction whereas the UPS does the opposite function in a three-step process involving 3 different classes of ubiquitinases. Therefore, DUBs can be more specific drug targets than UPSs.Since DUBs and UPSs control so many cellular functions involving their target proteins of various metabolic pathways infectivity of the parasite during host-parasite infection will likely be highly dependent on these proteases and their effect on the host immune functions (Ponder and Bogyo, 2007; Kim et al., 2014). Drug resistance against chemotherapeutic agents and the lack of private funding and R&D-based research for a cure of parasitic diseases in low-income countriesdemands alternatives. Here the role of DUBs can be of utmost interest since they are of different classes with function and provides the option of multi-stage drug development processes involving the 20S proteasome which is different from the human 26S proteasome complex (Alksne, 2002; Ponder and Bogyo, 2007). In this review, we focussed exclusively on parasite DUBs of which some of them are characterized earlier and some of them are putatively identified by us using *in silico* methods. We also explained the available data where the possible role of deubiquitination was mentioned although no functional studies of any of the DUBs were done independently in parasites yet. Based on the existing data on proteasome complex as drug targets we tried to rationalize how DUBs can be of importance for antiparasitic drug development.

## Mechanism of ubiquitination/deubiquitination cycle

**Figure 1:**
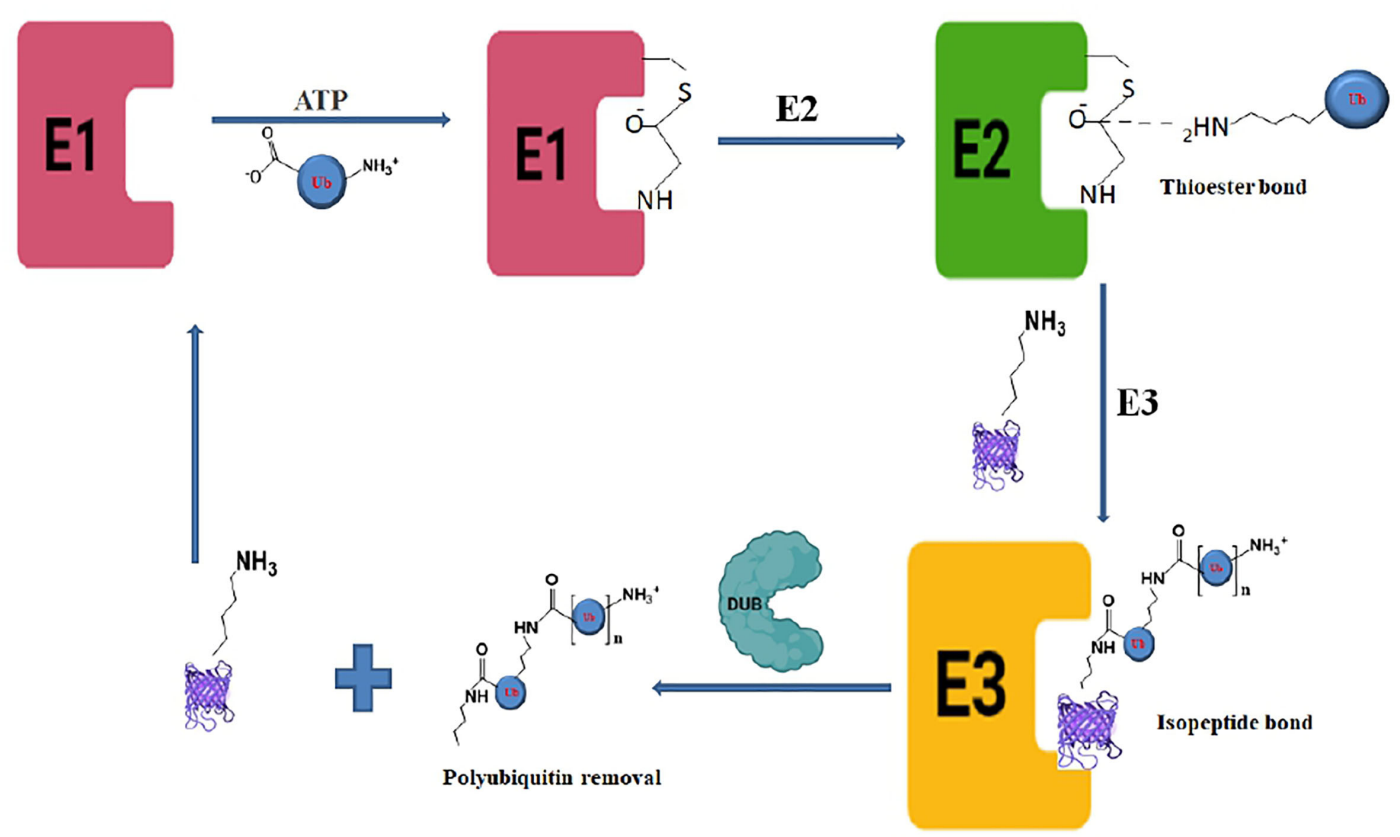
Biochemical pathway of ubiquitination and deubiquitination process showing the deubiquitinase enzyme activity in the last step.

**Figure 2:**
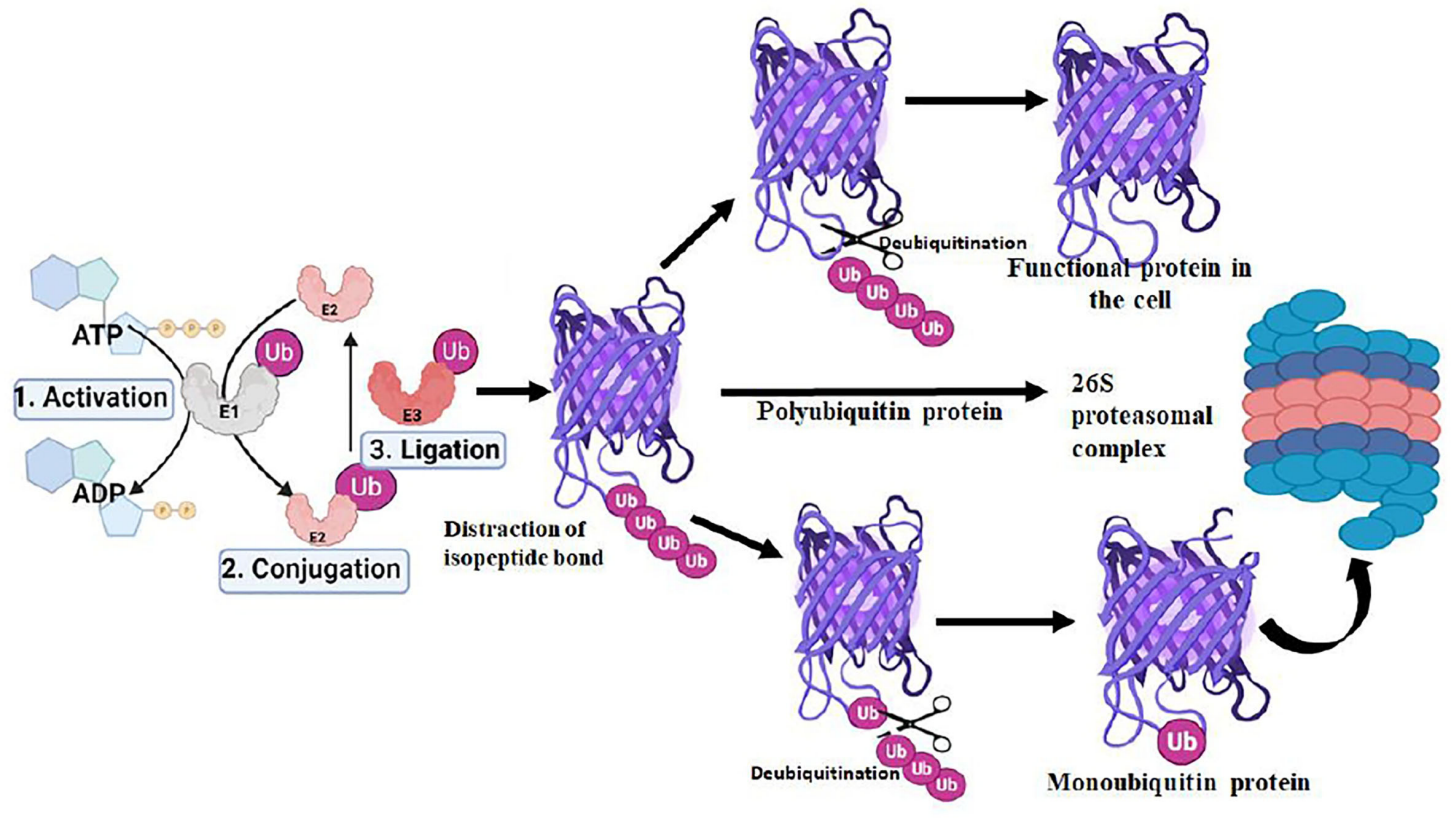
Role of deubiquitination in proteasomal degradation pathway of protein in the proteasome complex.

The ubiquitination process requires three enzymes: ubiquitin-activating (E1), conjugating (E2), and ligases (E3), which involve the transfer of ubiquitin molecules onto the target proteins that are eventually taken by the proteasome complex for degradation into peptides and amino acid residues (**Figure 1**) (Ebner et al., 2017; Deol et al., 2019).The E1 enzyme activates a ubiquitin molecule, which starts the process of ubiquitin coupling (Huang and Dixit, 2016). Ubiquitin C-terminus and cysteine residue of the E1 enzyme active site formed an ATP-dependent thiol ester bond through this step. A thioester-associated E2-ubiquitin intermediate is then used to allocate ubiquitin to the E2 enzyme. The E2 ubiquitin intermediate binds and interacts with the E3 enzyme which catalyzes the transfer of ubiquitin to the lysine present in the targeted protein. Finally, the 26S proteasome (Ronau et al., 2016; Liwocha et al., 2021) hydrolyzes the polyubiquitinated protein targets in an ATP-dependent manner (**Figure 2**). The process of monoubiquitination involves attaching a single ubiquitin molecule to one lysine residue in the substrate, whereas polyubiquitination involves attaching a chain of ubiquitin molecules to a specific lysine residue in the substrate. Monoubiquitination is typically used for DNA repair, vesicle sorting, signal transduction, and endocytosis (Sigismund et al., 2004; Sun and Chen, 2004; Sadowski et al., 2012; Ramanathan and Ye, 2012), Polyubiquitination, on the other hand, is mostly employed for protein deprivation and cell signaling (Komander and Rape, 2012). Depending on the substrate, ubiquitin chains can be organized in different ways, resulting in a variety of outcomes. For instance, protein activation and signaling pathways regulation have been linked to monoubiquitinationandlysine-63 polyubiquitination. Polyubiquitination induces proteasomal breakdown of substrates containing lysine-6 and lysine-48 (Ikeda and Dikic, 2008). The ubiquitination mechanism regulates the cell cycle, oncogenesis, immune responses, regulation of gene expression, apoptosis, and cell signaling pathways (Amerik and Hochstrasser, 2004).

The deubiquitination process involves the removal of ubiquitin molecules from the ubiquitinated-protein substrates and the post-translational changes induced in the ubiquitination process can be reversed by DUBs. The last step of ubiquitination where E3 ligases catalyze the protein ubiquitination can be reversed by DUBs to prevent protein breakdown. Although, the ubiquitination steps are ATP-dependent deubiquitination is not an ATP-dependent process. DUBs bind to ubiquitin-based isopeptide bonds, inhibiting the function of ubiquitin-protein ligases. DUBs are proteases and have four key biological functions including ubiquitin precursor processing, recycling, chain editing, and conjugation reversal (Amerik and Hochstrasser, 2004; Reyes-Turcu et al., 2009). As the crucial regulators of the UPS, therefore, DUBs play an important role in a variety of cellular processes, such as DNA repair, cell cycle progression, gene expression, and apoptosis. DUBs attack the carbonyl group of the Ub substrate isopeptide bond with a nucleophilic attack leading to the removal of ubiquitin chains for recycling in the UPS pathway. Based on the sequence motif, structural fold, and mode of action, DUBs are classified into major six distinct subfamilies based on their abundance. These include Ubiquitin-Specific Proteases (USP), Ovarian Tumor Proteases (OTU), Ubiquitin C-terminal Hydrolases (UCHs), JAB1/MPN/MOV34 (JMM)metalloenzymes, Machado-Josephin Domain proteases (MJDs), Motif Interacting with Ub-containing Novel DUB family (MINDY), and recently identified Zn finger with UFM1(Ubiquitin‐fold modifier 1) Specific Peptidase domain protein (ZUFSP). Any abnormality in ubiquitin proteasome pathway (UPP)-dependent protein ubiquitination is regulated by DUBs, inversely, thereby contributing to cellular homeostasis and controlling a variety of diseases, including cardiovascular & neurodegenerative disease, cancer, and systemic autoimmunity (Ambroggio et al., 2004; Komander et al., 2009).

## Different DUB domains as anti-cancer targets

In Environmental stress or endogenous cues, cells adjust their internal cellular functions by altering the abundance or activity of their proteins by post-translational modification (PTM) (Song and Luo, 2019). Ubiquitination is a part of proteins PTM which regulates the homeostasis of many biochemical pathways of the cell. The DUBs containing USP domains like USP7 and USP14 are the most well-studied since they are structurally validated. USP7 and USP14 are found to be associated with cancer and neurodegenerative diseases. Mutations in E3 ligases and lack of DUB activity with subsequent degradation of tumor suppressors are linked with malignant tumors (Kors et al., 2019). Inhibitors of E3, E2, E1, and DUBs are coined as potential molecular targets of cancer. DUBs inhibitors, sP5091 and P22077, were found to inhibit USP7 and effective in inducing apoptosis in malignant myeloma cells, which were resistant to conventional and bortezomib therapies in mouse tumor model studies (Deng et al., 2020). The chalcone derivatives G5 and F6 induced the Bcl-2-independent apoptosis to kill the cancer cells and were found to be broad spectrum inhibitors of DUBs (Aleo et al., 2006). Pimozide, a specific USP1 inhibitor, was found to block glioma stem cell maintenance and radio-resistance (Lee et al., 2015). WP1130, a small molecule was found to be an inhibitor of several DUBs including USP5, USP14, UCHL5, and UCH37. Furthermore, by downregulating the anti-apoptotic protein MCL-1 and upregulating the proapoptotic protein p53, it leads to anti-tumor activity (Karpiyevich et al., 2019). These findings and other studies provide ample evidence that DUBs can be anti-cancer targets in humans without affecting normal cells. Recently, Nelson et al. used established anti-cancer DUBs inhibitors for activity against *Plasmodium*. These small molecule inhibitors (PR-619, P5091, TCID, WP1130, b-AP15, NSC-632839) and 1,10 phenanthroline compounds (Altun et al., 2011; Simwela et al., 2021) showed synergism in action with artemisinin (ART) along with overcoming the ART-resistance for these parasites (Simwela et al., 2021). Inhibition of different USP and UCLH domains of DUB was coined as a factor for this antimalarial effect. These findings suggest that DUBs may be useful in the development of drugs to treat diseases other than cancers.

## Functional role of important DUB domains in human

### Ovarian tumor-related proteases

The OTU domain was first identified in the ovarian tumor gene of *Drosophila melanogaster* and that’s how this domain nomenclature is received (Makarova et al., 2000). The structural evaluation revealed that it belongs to the cysteine protease class and has a characteristic catalytic triad. It also has a specific recognition (Fiil et al., 2013) feature for the ubiquitination chain, for example, OTUB2 favors the substrate K63 di-ubiquitin (Altun et al., 2015). Only chains connected by K48 are broken by OTUB1 (Edelmann et al., 2009). Members of the OTU family are likely to recognize target substrates because of their ability to distinguish between polyubiquitin chains with various degrees of chain length. Deregulated A20 deubiquitinase activities are linked to inflammatory and autoimmune disorders. A20 is an OTU domain-containing protein that modulates NF-κB activation and signaling. Interestingly, A20 gene variants have been detected in autoimmune disorders and human lymphoma (Majumdar and Paul, 2014).In OTU-domain containing DUBs, the proteolytic catalytic trio is made up of conserved Cys, His, and Asp residues. The OTU protease family includes members in which the OTU-related motif is part of a ubiquitin-specific processing proteases family protein, indicating a link for deubiquitination in chromatin remodeling involving histone proteins as targets (Atanassov et al., 2011).These DUBs have also been related to a variety of neurological and immunological diseases, as well as inflammation involving microbial infections.

### Ubiquitin-specific processing enzymes

In the active site of the catalytic USP domain, the catalytic triad is a feature shared by all USP members, which are cysteine proteases. The thumb, palm, and fingers of a human right hand were first compared to the USP7/HAUSP-herpesvirus-associated ubiquitin-specific protease structure (Pozhidaeva and Bezsonova, 2019) when describing the three sub-domains that make up the conserved USP domain. The finger sub-domain interacts with the ubiquitin substrate, and the palm, thumb, and active site resemble the catalytic triads as found there (Hu et al., 2002). A catalytic domain with accessory domains is frequently seen in DUBs, some of which aid in target detection (Kim et al., 2016).USPs are the largest amongst all DUBs subfamily that contributes to the development of various malignancies (Clague et al., 2019; Li et al., 2022). Therefore, more research using these DUBs as a therapeutic target is necessary. USP14 is suspected to play a role in ovarian and colorectal cancers, among other malignancies. USP14 appears to play a function in ovarian cancer cell line screening for genetic abnormalities and 3T3 focal formation assays (Wada et al., 2009). Elevated USP14 expression was connected to clinical stages with liver and lymph node metastases in another investigation of colorectal cancer patients (Maiti et al., 2011). Bioinformatics studies have shown that the USP14 domain of DUB protein in humans is different from parasites by including two short but well-conserved motifs known as the Cys and His boxes, which contain all of the catalytic triad residues as well as other residues in the active site pocket (Shinji et al., 2006). Recent research has shown that the USP20 domain plays a crucial role in the carcinogenesis of several cancer types, including adult T-cell leukemia, breast cancer, colon cancer, lung cancer, and gastric cancer. Consequently, modulating USP20 activity can be of unique cancer therapy (Li et al., 2022).

### Ubiquitin C-terminal hydrolases

UCHs, including UCHL1, UCHL3, UCHL5/UCH37, and BAP1(BRCA-1 Associated Protein-1), are also cysteine proteases. Typically, substrates of these DUBs are tiny protein fragments like short polypeptides or protein domains. Large ubiquitinated proteins cannot be bound or catalyzed to the same extent as smaller ubiquitinated proteins due to the small and tight pocket on the active site of UCHs and the restriction of the loop diameter (Bishop et al., 2016; Lou et al., 2016). The UCHs and many USPs share a similarly three-dimensional design and a superimposable catalytic triad, however, they differ in the amino acid sequence of the catalytic positions (Amerik and Hochstrasser, 2004; Mondal et al., 2022 126(1). The sequence similarity of UCHL1 DUBs is limited to the domain containing the catalytic triad of Cys, His, and Asp/Asn residues. The rest of the protein sequence differs greatly amongst USPs, and this difference is assumed to be critical for proteins/peptides as substrate recognition. Whether the S18Y allele for UCHL1 protects against sporadic Parkinson’s disease or is a risk factor for it, has been the subject of debate in several studies (Ragland et al., 2009; Miyake et al., 2012). A crucial DUB is UCHL3 (Ubiquitin carboxyl-terminal hydrolase L3), a member of the UCL subfamily. Overexpression of UCHL3 in breast cancer cells makes them resistant to radiation and chemotherapy, while depletion of UCHL3 reverses the phenomena indicating a role of UCHL3 in cancer therapy. Here, UCHL3-mediated deubiquitination of RAD51 protein, important in homologous recombination, was found to recruit BRCA2 protein, the key regulator of breast cancer (Luo et al., 2016). It is also observed that UCHL3 protects the Forkhead box M1 (FoxM1) protein which regulates the transition from the G1 to S phase in the cell cycle. The overexpression of UCHL3 is investigated in pancreatic cancer leading to regulation in the cell cycle through FoxM1 (Song et al., 2019). A UCH type of protease is ubiquitin C-terminal hydrolase L5 (UCHL5)/Uch37 which can prevent the proteolysis of weakly ubiquitinated proteins and cleave the Ub from the distal end of chains of poly-ubiquitination (Chen and Walters, 2015). UCH37 plays a role in embryo development because mice with the UCH37 gene knock-out died of severe defect during embryonic brain development (Al-Shami et al., 2010). According to Da Liu et al., UCHL5 contributes to the development of endometrial cancer by activating the Wnt/β-Catenin Signaling Pathway (Liu et al., 2020). All these reports suggest that UCH domain-containing DUBs could be vital anti-cancer targets.

### Machado–Joseph disease proteases

Currently, there are four members of the MJDs/Josephin family: ATXN3/ataxin3, ATXN-3L, JOSD-1, and JOSD-2 in human are known. A catalytic triad is found with one cysteine and two histidine residues that are highly conserved (Burnett et al., 2003). Human *ataxin-3*, a DUB, is involved in Machado-Joseph disease (MJD). The mutant *ataxin-3* was involved in this type of polyglutamine neurodegenerative disease although the mechanism is unknown (do Carmo Costa and Paulson, 2012).

### Motif interacting with Ub-containing novel DUB family (MINDY)

Members of the MINDY family, which in humans includes MINDY-1, -2, -3, -4, and -4B, are only found on K48-linked ubiquitin chains (Rehman et al., 2016). The protein fold of MINDY-1 in its crystal structure was unique and had no resemblance to any other kind of DUB (Rehman et al., 2016). Long polyubiquitin chains are preferred by MINDY-1, which also cleaves the distal ubiquitin component. The catalytic triad is composed of cysteine, glutamine & histidine and the active site in MINDY-1 is modified upon substrate binding from its non-productive conformation in the absence of the substrate (Kristariyanto et al., 2017).

### Jad1/Pad/MPN domain-containing metalloenzymes and zinc finger with UFM1-specific peptidase domain

The JAMMs are the only family of metalloprotease-related DUBs that have a Zn atom in the active site with a Glu-x[N]-His-x-His-x (D'Arcy et al., 2015)-Asp motif which coordinates the binding of two Zn^2+^ ions. The human genome encodes 14putative JAMM DUBs, in this catalysis required nucleophilic attack by DUBs to the carbonyl group of Ub (Komander et al., 2009; Suresh et al., 2020). Human ZUFSP selectively interacts and cleaves long K63-linked ubiquitin chains using tandem ubiquitin-binding domains, but displays poor activity toward mono- or di-Ub substrates (Hermanns et al., 2018).

## Functional role of DUBs indifferent parasites

### Schistosoma

**Figure 3:**
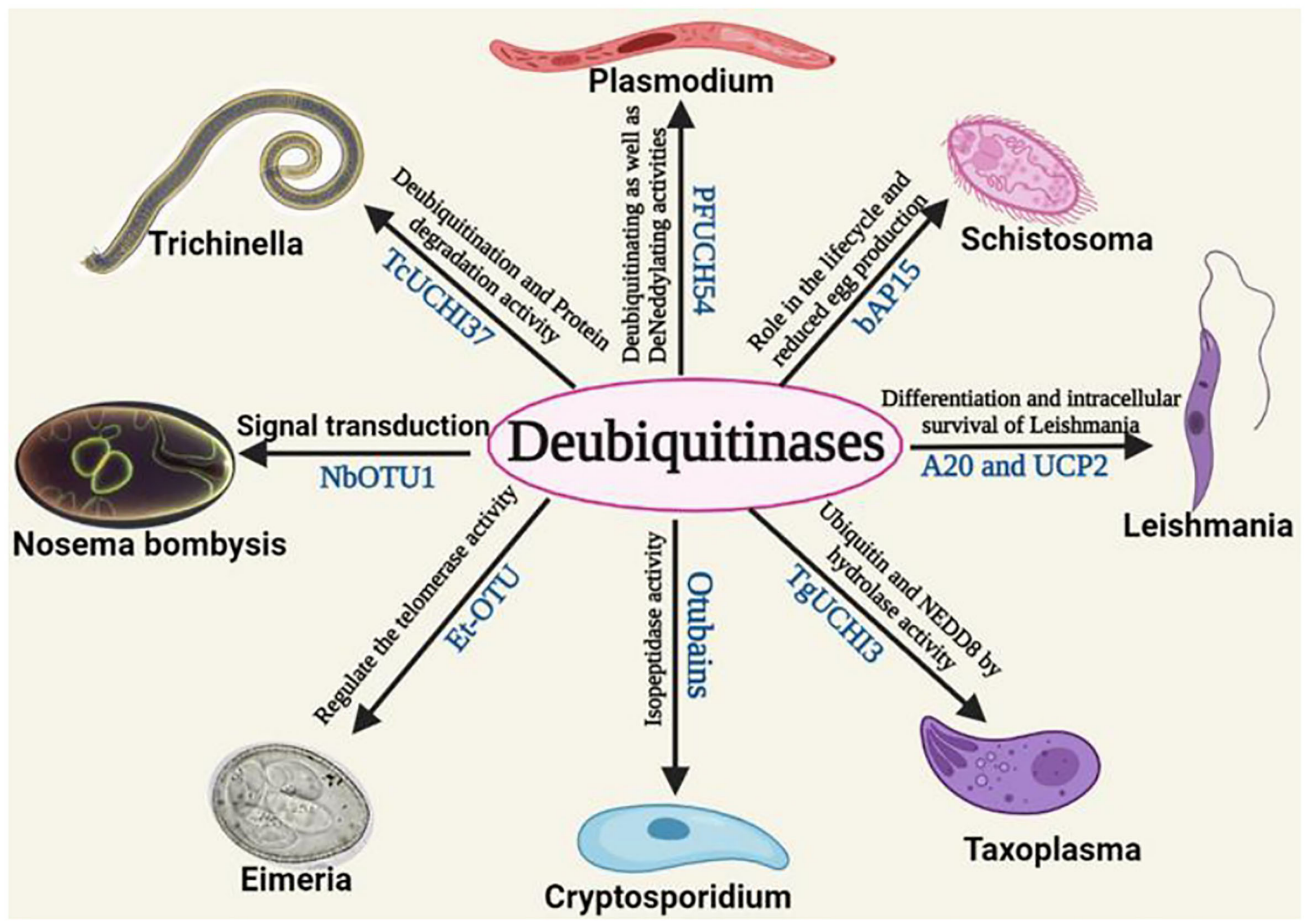
The role of different deubiquitinases with their respective domains showing different functions in the parasites.

All the functional role of DUBs of different parasites is mentioned in **Table 1**. Schistosomiasis, also known as bilharzia, is caused by Schistosomes, a parasitic worm. It is the second most fatal parasitic disease in humans, after malaria. Humans are mostly affected by *Schistosoma mansoni*, *Schistosoma haematobium*, and *Schistosoma japonicum*. Praziquantel, a prescription medicine, is generally used to treat both schistosome infections (Kubes and Jenne, 2018). Schistosomes must undergo a variety of morphological and metabolic changes to adapt and grow in the host vertebrate body (Stirewalt, 1974; Fishelson et al., 1992). These modifications need epigenetic changes involving a large number of proteins. The *S. mansoni* genome contains the MJD and OTU domain-containing proteins. These proteins were found to share significant similarities with human proteins from the same families. These genes were also found to be differentially expressed during the parasite *S. mansoni’s* various life cycle stages indicating its possible role in virulence and/or survival. The structure of MJDs was similar to that of human *ataxin-3*, with 55.56% and 29.33% identity in sequences with *S. mansoniataxin-3* and *S. mansoni* Josephin. Phylogenetic analysis has also shown that these proteins and OTUs of humans are conserved in subfamily and are orthologous to *S. mansoni* species. Different OTUs of human and *S. mansoni* including Otubain, OTU1, OTU3, OTU5a, and OTU6b have shown to have significant structural similarity with 45.13, 49.70, 40.40, 48.54, and 21.66% sequence similarity for these DUBs, respectively, between these two species (Pereira et al., 2015a). Similarly, other categories of DUB domains such as UCH and USP have been found to be regulated and differentially expressed during the development of *S. mansoni.* It aids in the cellular process involving egg production of worms. All DUB domains are conserved among all *Schistosoma* species (do Patrocinio et al., 2020). A non-selective DUB inhibitor (PR-169) was evaluated in 2021 by Andressa et al. for *Schistosoma*. PR-169 inhibition involves apoptosis, autophagy, and the transforming growth factor beta (TGF-β) signaling to lead to changes in parasite oviposition thereby not allowing them to lay eggs properly (Barban do Patrocínio et al., 2021). Therefore, these DUBs of *Schistosoma* could be potential drug targets (Pereira et al., 2015b). In **Figure 3**, the role of different deubiquitinases with their respective domains showing different functions in the parasites were shown.

**Table 1:** Functional role of DUBs in different parasites.

Species Name	GenbankID/UniProtKB/VEuPathDB	Class (DUB domain)	DUBs	Function for Drug target	Ref.
*Schistosoma mansoni*	XP_018650214.1	MJD	Ataxin-3	Involved in life cycle	(Stirewalt, 1974), (Fishelson et al., 1992)
*Schistosoma mansoni*	XP_018645017.1	USP	USP14	It found in different life cycle stages indicating their involvement in cellular processes required for *S.mansoni* development	(Pereira et al., 2015a), (do Patrocinio et al., 2020)
*Trichinella Spiralis*	157958881	UCH	UCHL5	Deubiquitination activities	(Song et al., 2018)
*Trichinella Spiralis*	13182314	OTU	OTU68	Protein degradation	(White et al., 2011)
*Plasmodium falciparum*	Gene - PfNF54_110021400	UCH54/NF54	PfUCH54	Deubiquitinating as well as deNeddylating activities	(Artavanis-Tsakonas et al., 2006)
*Plasmodium falciparum*	Gene - PfNF135_140064500	UCHL3	PfUCHL3	Essential for the survival of the parasite	(Frickel et al., 2007)
*Plasmodium falciparum*	Gene - PF3D7_1031400	OTU	mOTU	It helps in the entry of plasmodium into the host cell	(Hunt et al., 2007)
*Toxoplasma gondii*	XP_002365447.1	UCH	TgUCHL3	Deubiquitinating as well as deNeddylating activity and protein degradation, the cell cycle and transcription progression of the cell cycle	(De Monerri et al., 2015)
*Toxoplasma gondii*	A0A7J6K192	OTU	TgOTUD3A	Plasticity of apicomplexan cell cycle architecture.A novel regulator of dendritic cells during infectious and inflammatory diseases.	(Mulas et al., 2021) (Dhara et al., 2017),
*Eimeria* *tenella*	XM_013374305.1	OTU	Et-OTU	Expression at different phase of Eimeria *tenella* life cycle and also regulate *E. tenella* telomerase activity	(Wang et al., 2018)
*Cryptosporidium parvum*	XM_001388292.1	OTU	CpOTU	Phathophysiological role in oocyst stage of the parasite and isopeptidase activity.	(Ju et al., 2014)
*Leishmania infantum*	A4HXL8	OTU	OTULi	Proinflammatory response in stimulated murine macrophages	(Azevedo et al., 2017)
*Trypanosoma cruzi*	A0A7J6YC36	COP9	–	Cell cycle regulation.	(Ghosh et al., 2020)
*Nosema bombycis*	R0KME3	OTU	NbOTU1	Protein degradation, signal transduction, cell immune response, and deubiquitination activity *in vitro*.	(Wang et al., 2015)

### Trichinella

*Trichinella spiralis* is a zoonotic food-borne parasite of phylum nematode. It is mainly responsible for the disease trichinellosis which is caused due to eating raw or uncooked meat infected with these parasites. Infections caused by the parasite in humans range from mild flu-like symptoms to myocarditis, encephalitis, and death. The severity of the disease is determined by the infecting worm population (Mitreva and Jasmer, 2010). *T. spiralis* larvae cause chronic infections in immunocompetent hosts’ skeletal muscles. This is also essential for the parasite’s transmission and survival in nature. Apart from that, at all phases of infection, it modulates host immunity as well as normal cellular and subcellular functions (Wakelin, 1993; Fabre et al., 2009; Mitreva and Jasmer, 2010). It has been found that the cysteine proteinase of *Trichinella* plays a very important role in the larval invasion, survival, and development of *T. spiralis*. Further, using the antibodies against this protein and immunofluorescence studies it was confirmed that this protein is present in the parasite cuticle and expressed at all stages of parasite life. It is, therefore, really important to study and characterize other cysteine proteases in *T. spiralis* (Song et al., 2018). To date, only one deubiquitinase enzyme has been characterized in *T. spiralis*. The characterized DUB TsUCH37, a cysteine proteinase, was found to share significant sequence similarities with human UCH-L5, implying that the proteasome-DUB interaction is conserved throughout evolution across different species. The UCH inhibitor LDN-57444 reduced the deubiquitinase activity of recombinant TsUCH37 and reduced the viability of cultured larvae. Therefore TsUCH37 can be a potential drug target in *Trichinella* (White et al., 2011).

### Plasmodium

The most lethal form of human malaria is caused by the blood-borne parasite *P. falciparum*, which kills up to 2 million people per year globally (Kusotera and Nhengu, 2020). The continuous evolution of resistant strains demands the development of innovative strategies to overcome the global malaria problem where effective vaccinations are not developed yet (Wang et al., 2009; Menard and Dondorp, 2017).In all eukaryotic cells, the UPS controls damaged protein levels by covalently modifying protein substrates with ubiquitin and then directing them to the proteasome for degradation including *Plasmodium* (Pickart and Cohen, 2004).In the genome of *P. falciparum*, about 17 DUBs have already been discovered *(* Ponts et al., 2008 *).*. Using bioinformatics analysis, approximately 29 DUBs in *P. falciparum* have been identified, though the activities of these putative enzymes need to be investigated (Artavanis-Tsakonas et al., 2006).The first DUB discovered in the *P. falciparum*, is PfUCH54 which is also known as UCH37/UCHL5 in human UPS, and was found to have deubiquitinating and deNeddylating activities (Frickel et al., 2007). The post-translational modification neddylation is very similar to ubiquitination except that it is carried out by a protein called NEDD8. However, there are just a few neddylation processes known so far (Rabut and Peter, 2008). Neddylation is carried out by the NEDD8 protein which also functions as an E3 ubiquitin ligase, although neddylation is conserved among eukaryotes, little is known about this protein in *Plasmodium* and other apicomplexan parasites. Without a C-terminal tail, PfNEDD8 is a 76 amino acid residue protein, making it easily conjugatable (Rabut and Peter, 2008; Bhattacharjee et al., 2020). *Toxoplasma gondii* and *P. falciparum* also have conserved regions of UCHL3, and PfUCHL3, which have ubiquitin and Nedd8-related activities. PfUCHL3 has also been discovered to be important for the parasite’s survival as well as for parasite entry into the host cell and could be a potential drug target in the future (Siyah et al., 2021). In addition, drug resistance in *Plasmodium* has been linked to a locus containing a UBP-1 gene, which encodes a DUB homologous to USP7 (Hunt et al., 2007).In the battle against the plasmodium parasite, researchers are now focusing their efforts on identifying new drug targets for the DUB enzymes. Currently, the potency of small molecules against the DUB enzyme of *P. falciparum* was evaluated and it has shown efficacy to inhibit intra-erythrocytic maturity of malaria parasites *in vitro*. It also showed the data related to 17 DUBs with proposed functionality and essentiality of these DUBs in the genome by an *In-silico* study. Inhibitors that target the proteasome, which is a critical component of the UPS, show activity against malaria parasites and work in tandem with artemisinin. They found that small molecule inhibitors of mammalian DUBs were effective against *Plasmodium* parasites (Simwela et al., 2021). Using a functional chemical approach, it was shown that deNeddylation is controlled by a different set of enzymes in the parasite compared to the human host, indicating the possible role of deubiquitination/deNeddylation of PfUCH37 as a possible drug target (Karpiyevich et al., 2019).

### Toxoplasma

*Toxoplasma gondii* is a parasite that causes toxoplasmosis in humans and other animals, including dogs, cats, sheep, and goats. Immune-compromised living creatures are primarily exposed to these symptomatic infectious diseases. The three primary causes are congenital infection, ingestion of infected tissues, and oocyst ingestion. *T. gondii* infection is acquired transplacentally in fewer than 1% of cases (Hill et al., 2005; Dubey and Jones, 2008).Post-translational modifications related to protein ubiquitination play crucial roles inside the parasite body. *T. gondii’s* genome was discovered to encode a large number of proteins involved in the ubiquitination/deubiquitination machinery (De Monerri et al., 2015). *T. gondii* encoding TgUCHL3, an orthologous of human UCHL3, was the first active deubiquitinase that was found to have dual ubiquitin and NEDD8 hydrolase activity (Frickel et al., 2007). TgOTUD3A is a *T. gondii* deubiquitinase that belongs to the OTU family. TgOTUD3A is a cytoplasmic protein that is low in the G1 phase of the parasite cell cycle but its expression increases as the cell cycle progress. TgOTUD3A was found to extract ubiquitin from the K48-linked chain of ubiquitin but not from the K63-linked ubiquitinated substrate. This order for specific lysine linkages (K48 > K11 > K63) was polyubiquitination chain specific. The TgOTUD3A-KO mutant sheds light on the mechanisms that underpin apicomplexan cell cycle architectural plasticity. The deubiquitinase OTU domain, ubiquitin aldehyde binding 1 (OTUB1) is upregulated in dendritic cells upon murine *T. gondii* infection and lipopolysaccharide challenge. The DUB (OTUB1) increases NF-κB-dependent immune responses in dendritic cells during infection or inflammation by stabilizing UBC13 (Mulas et al., 2021).

### Eimeria

The protozoan parasite *Eimeria* is responsible for the worldwide spread of chicken coccidiosis. It is an intestinal caecal lesion disease that is regarded as one of the most economically damaging diseases of domestic poultry (Allen and Fetterer, 2002). *Eimeria acervulina*, *Eimeria maxima*, *Eimeria mitis*, *Eimeria praecox*, *Eimeria brunetti*, *Eimeria necatrix*, and *Eimeria tenella* are seven species of *Eimeria* that infect domestic chickens and cause malabsorptive or hemorrhagic enteritis. Three species, *E. acervulina*, *E. maxima*, and *E. tenella*, are most common in young chickens and become highly pathogenic when they mature (Clark et al., 2016). Several cysteine proteases related to the UCH and OTU domain family of deubiquitinase enzymes have shown their expression at different phases of the *E. tenella* life cycle (Ma et al., 2021). RNA-dependent RNA polymerase of *E. tenella* was found to be associated with the deubiquitinating enzyme of the OTU domain family protein of the host. These two proteins interact with each other at both intracellular and extracellular levels. Et-OTU can also remove Lys-48 and Lys-6linked di-ubiquitin substrates *in vitro*, but not from Lys-63, -11, -29, and -33 linked di-ubiquitin chains (Wang et al., 2018). Deubiquitinase from *E. tenella* ovarian tumor (Et-OTU) was previously found to control telomerase function. *E. tenella* RNA virus 1-RNA-dependent RNA polymerase (Etv-RDRP) interacts with Et-OTU and enhances its deubiquitinating function, while also helping to develop oocyst walls. Similarly, in *E. acervulina*, a novel functional deubiquitinase OTU domain family was identified and shown to be highly linkage-specific, cleaving Lys48, 63, and 6 linked deubiquitination chains (Wang et al., 2019).

### Cryptosporidium

*Cryptosporidium* is a parasite that causes cryptosporidiosis, a sickness that affects both animals and humans. Only a few of the species are pathogenic to human beings. *Cryptosporidium* can be transmitted in many ways, but the most common way to spread the parasite is through water (drinking and recreational water). The parasite’s outer shell protects it from chlorine disinfection and allows it to exist outside the body for long periods (Zaheer et al., 2021). *Cryptosporidium parvum* can stop an infant’s development suddenly and can be fatal in immunocompromised people. The anti-parasitic medications nitazoxanide and paromomycin are most commonly used to treat cryptosporidiosis, albeit both have limited efficacy. A cysteine protease inhibitor, N-methyl piperazine-Phe-homo Phe-vinyl sulfone phenyl (K11777), was found to inhibit the growth of *C. parvum*-infected mammalian cell lines (Ndao et al., 2013). The inhibitor also showed high efficacy with no toxicity in the parasite-infected C57BL/6 gamma interferon receptor knockout (IFN-γR-KO) mouse model, which is highly susceptible to *C. parvum* infection. The oral or intraperitoneal treatment with K11777 for 10 days (210 mg/kg of body weight/day) rescued mice from otherwise lethal infections of this parasite. Bioinformatics analysis followed by analysis with recombinant cryptopain 1 (a cysteine protease with deubiquitinase activity) from the parasite showed that K11777 strongly binds to this enzyme, and, therefore inhibits the growth of this parasite *in vivo*. Otubains, a cysteine protease family that includes other cysteine proteases, play a significant role in the ubiquitin pathway. In *C. parvum*, a protein from this family was partially characterized and found to have deubiquitinating activities. The amino acid aspartate, cysteine, and histidine residues that make up the catalytic triad of otubains were found in the gene encoding otubain-like cysteine protease of *C. parvum* (CpOTU). It possesses isopeptidase activity at neutral pH with a pathophysiological role in the parasite’s oocyst stage (Ju et al., 2014). Further, CpOTU had an unusual C-terminal extension of 217 amino acids which is essential for the activity of the enzyme but absent in mammalian counterparts. This suggests that DUBs of similar kinds in parasites can be explored as a drug target.

### Leishmania

Leishmaniasis is caused by more than 20 different types of *Leishmania*. Leishmaniasis is currently a major problem in more than 90 tropical and subtropical regions since it is a neglected tropical disease (NTD). A million new cases are estimated to be registered annually. Sandflies are carriers of the parasites (genus *Phlebotomus*). When an infected sandfly feeds on blood, it injects promastigotes into humans. These promastigotes either deliberately invade macrophages or are phagocytosed by them. Promastigotes within macrophages differentiate into amastigotes. The amastigotes multiply within macrophages by binary fission and can infect additional cells (Alvar et al., 2012). The disease can manifest in three ways, depending on the species and environmental factors: visceral leishmaniasis (VL), cutaneous leishmaniasis (CL), and mucocutaneous leishmaniasis (MCL). VL affects the spleen and liver mainly, and, can be fatal if left untreated (Sundar and Chakravarty, 2018).

Deubiquitinase enzymes play several important roles in controlling severalcellular events of this eukaryotic parasite. The genome of *Leishmania infantum* contains 27 predicted DUB proteins, 16 belong to USP, 5 to JAMM, 4 to OTU, and 2 to the UCH family. Based on its low amino acid identity, compared to human otubain-1 (33%) and otubain-2 (26%), the otubain, a protein from the OTU domain family of *Leishmania* DUB, may be a new therapeutic option for leishmaniasis. Otubain of *L. infantum*(OtuLi), which was prepared recombinant along with three mutants with point mutation on OTU domains, showed activity on lysine 48 (K48)-linked tetra-ubiquitin over K63-linked tetra-ubiquitin (Ub) as substrate. Treatment of macrophages, *in vitro*, with OtuLi, was found to induce inflammatory responses in macrophages by causing substantial TNF- and IL-6 secretion. OtuLi is an enzyme that resides in the cytoplasm of the parasite, has a preferential K48-linked substrate specificity, and the residue F82 was found critical for deubiquitinase activity (Azevedo et al., 2017).Even though DUB enzymes play various important roles in the life cycle of *Leishmania* parasite, *Leishmania* can hijack the host’s protective strategies by manipulating the TLR signaling pathway and interacting with the host’s deubiquitinating enzyme A20 (Azevedo et al., 2017; Gupta et al., 2017). Short hairpin RNA-mediated knockdown of A20 and mitochondrial uncoupling protein 2 (UCP2) in *Leishmania donovani*-infected mice independently documented decreased liver and spleen parasite burden and increased IL-1β production. These results suggest that *Leishmania* exploits host A20, which restricts deubiquitination of pro IL-1β (Duong et al., 2015), and UCP2 to impair inflammasome activation for disease propagation. Activity-based protein profiling of parasite *L. Mexicana* revealed that the deubiquitinating cysteine peptidases (C12, C19, and C65) activity remain relatively constant during differentiation from procyclic promastigote to amastigote. However, CrispR-Cas9-based generation of null mutants showed that DUBs 1, 2, 12, and 16 are essential for promastigote viability and DUB2 is essential in establishing parasite infection to the host. This study shows that DUB2 is in the nucleus and interacts with nuclear proteins associated with transcription/chromatin dynamics, Further, DUB2 has broad substrate specificity, cleaving all the di-ubiquitin chains except for Lys27 and Met1, *in vitro*. Therefore, *L. Mexicana* DUB2 and its homologs in other *Leishmania* can be used as suitable drug targets (Damianou et al., 2020).

### Trypanosoma

Millions of people in 36 countries in Sub-Saharan Africa are at risk of sleeping sickness. The illness is considered fatal if not treated and the disease is transmitted by tsetse fly. Depending on the parasite involved, human African trypanosomiasis can take two forms: More than 98 percent of confirmed cases are caused by *Trypanosoma brucei gambiense*. Nearly 40, 000 cases were documented in 1998, but it was estimated that 300, 000 cases went undiagnosed and thus untreated. For the first time in 50 years, the number of cases registered fell below 10,000 (9,878) in 2009, thanks to continued control efforts. With 997 new cases registered in 2018, the lowest amount since systematic global data collection began 80 years before, the decrease in cases is continuing. The population at risk is projected to be 65 million people. The shortage of new drug molecules necessitates the discovery of new targets for drug production (Shah, 2012).

TbPEX4 is present in procyclic PEX4 trypanosomes and it has a role in the cell cycle of *T. brucei (* Gualdrón-López et al., 2013). Different DUBs domains; WLM, OTU, JOSEPHIN, Peptidase_C48, Peptidase C54, Peptidase C97, UCH, JAB, Ribosomal_S19e are found in *Trypanosoma* species. Gupta et al., identified new drug targets against new DUB domains (WLM, and Peptidase C97) in *Trypanosoma cruzi* which are absent in humans (Gupta et al., 2018). When the parasite life-cycle changes from metacyclic trypomastigote to amastigote, ubiquitin-dependent degradation has been observed for cytoskeleton proteins. As a result, this phase of the life cycle can be targeted to prevent the degradation of cytoskeleton proteins, hence interfering with the parasite’s ability to survive inside the host. The number of UPP components found in *T.cruzi* is the largest of any parasite species. In another analysis, the constitutive photomorphogenesis 9(COP9) signalosome was found to be critical for parasite biology, as interruption of the parasite COP9 caused dysregulation of the UPP, impairing protein degradation and resulting in cell death (Ghosh et al., 2020). The COP9 signalosome (CSN) was found to be conserved in a variety of pathogens, including *Leishmania*, *Trypanosoma*, and *Toxoplasma*. This DUB pathway of trypanosome needs to be investigated further, and a new inhibitor for DUBs could be developed in the future.

### Nosema

Microsporidia is a group of intracellular parasites that are found in over 1200 different species throughout the world. It has the ability to infect all major animal lineages as well as humans (Williams, 2009).*Nosema bombycis* is a species of Microsporidia of the genus *Nosema* infecting mostly silkworms and responsible for pébrine disease. Immunocompromised mammalian individuals are more vulnerable to infections caused by this group of microorganisms (Didier et al., 1998). In microsporidia, the ubiquitination/deubiquitination proteasome pathway is poorly understood. Only one DUB protein belonging to the OTU domain family, which is conserved in most of the parasitic species, has been partially characterized. The *N. bombycis*, has deubiquitinase activity in a 25 KD a recombinant otubain-like protease (NbOTU1) against K48-linked tetraubiquitin substrate, *in vitro* (Wang et al., 2015). The expression of NbOtu1 has observed day 3 post-infection and immunofluorescence analysis indicated that NbOtu1 is localized on the spore wall of *N. bombycis*. The subcellular localization of the NbOtu1 further showed that NbOtu1 is localized in the regions around the endospore wall and plasma membrane. More research is required to understand about DUBs in *N. bombysis* which causes significant damage to the economically important insect, *Bombyx mori*.

## *In-silico* based screening of various possible DUB domains in parasites

The list of distinct DUB proteins was compiled by using the most recent Uniprot version and a search of the common domains (12 Pfam domains) found in DUBs of different parasites. The members of the numerous DUB families and subfamilies in the human genome served as models for the selection of hidden Markov models (HMMs). Using this model we analyzed the DUB domains in different parasites. There are variable numbers of putative DUBs present in different species of parasites. The highest number of DUBs was found in *S. mansoni* with 114 DUBs and in *N. bombyci*s with the lowest in number with 22 DUBs amongst all other parasites. The proteome of *Trypanosoma*, *Trichinella*, and *Toxoplasma* contain 77, 67, and 46 putative DUBs respectively. The other parasites have putative DUB proteins which are less than 40 based on our search. The low or variable numbers of DUBs in different parasites may suggest the incompleteness of the sequencing data of their respective genomes which is available to date. The UCH-domain containing DUBs are present in large numbers whereas Peptidase-C65 containing DUBs was very few in numbers among all the parasites. The OTU-domain containing DUBs were 38 in *S. mansoni*, the highest, as compared to all other parasites that contain the similar OTU proteins. The crystal structure of OTU1 and OTU2 in human has given a clear view of these DUBs deubiquitination mechanism and their difference from other DUBs (Nanao et al., 2004; Messick et al., 2008). The structure of a human OTU2 reveals a five-stranded β-sheet flanked by two α-helical domains that are novel for any DUB enzymes (Nanao et al., 2004)with a catalytic triad of Cys51-His224-Asn226. The human OTU1 is composed of 6 β-sheet cores surrounded by 3 α-helices where the catalytic triad is composed of Cys120-His222-Asp224residues.Otu1 binds with polyubiquitin chain analogs preferentially than monoubiquitin and hydrolyzes longer polyubiquitin chains with Lys-48linkages and not the Lys-63 or Lys-27 linked chains. These kinds of structural and functional studies of human OTUs with which *S. mansoni* are structurally similar may lead to the potential discovery of DUB inhibitors that can kill the parasites. The novel DUBsWss1p-like metalloproteases (WLM), Ribosomal_S19e and Mov 34 (JAMM family), and JOSEPHIN were present only in a few apicomplexan parasites and absent in the rest of the other parasites. All the detail of *In-silico* screened DUB proteins in different parasites are listed in **Table 2**.

**Table 2:** List of different DUBs domains and their numbers in all the parasites.

Hidden Markov models (HMM)	*Schistosoma mansoni*	*Trichinella spiralis*	*Plasmodium falciparum*	*Toxoplasma gondii*	*Eimeria acervulina*	*Cryptosporidium parvum*	*Leishmania donovani*	*Trypanosoma cruzi*	*Nosema bombycis*
WLM	–	–	1	1	–	–	1	2	–
OTU	38	2	2	9	6	3	2	6	3
JOSEPHIN	9	6	1	1	–	1	–	–	–
Peptidase-C12	11	4	2	2	2	2	2	–	–
Peptidase-C48	4	12	2	3	4	2	1	3	1
Peptidase-C54	5	2	2	1	1	1	2	2	–
Peptidase-C97	4	1	–	–	2	2	5	10	2
Peptidase-C65	1	1	–	1	1	–	1	2	3
UCH	29	28	10	16	12	9	15	31	10
JAB	10	11	–	6	5	3	4	14	3
Ribosomal_S19e	3	1	–	–	1	–	–	7	–
Mov 34	–	–	6	7	1	4	3	–	–
Total	114	67	26	46	35	27	36	77	22

## Most plausible DUBs as targets in the parasites

The functionally significant DUBs protein sequences of various classes were used as a query protein in a BLASTP search against the parasite databases provided in **Table 3**. Among all parasite proteins, *T. spiralis* protein shares the most identity with *Homo sapiens* USP (HsUSP14,96% query cover and 44.65% identity). However, parasites such as *T. cruzi*, *C. parvum*, *T. gondii*, and *L. donovani* were discovered to have DUBs almost similar to HsUSP14 protein (97-99% query cover and 30% to 33% identity). While against the HsUSP14, *S. mansoni* and *P. falciparum* was found to share the identity of 41.91% and 33.19% following query coverage of 77% and 87% respectively. In *N. bombycis*, the HsUSP14 homolog was found to be absent. Interestingly, human USP14 contains a total of seven phosphorylation sites of which the Akt-mediated USP14 phosphorylation at Ser432 activates its DUB activity and facilitates cleavage preferentially towards Lys48- and Lys63-linked chains rather than linear ubiquitin chains (Xu et al., 2015). This structural/functional information of human USP14-containing DUBs and their similarity with parasite DUBs may lead to the design of antiparasitic drugs targeting specifically parasite USP14 DUBs.

**Table 3:** Sequence Identity and query coverage by *in-silico* (BLAST) analysis of different identified DUB domains of parasites compared to humans.

Parasite	USP14			USP7			USP2a		
	Query coverage	% Identity	Accession ID	Query coverage	% Identity	Accession ID	Query coverage	% Identity	Accession ID
*Plasmodium falciparum*	87%	33.19%	ETW53368.1	44%	36.28%	ETW37327.1	53%	35.83%	ETW50834.1
*Schistosoma mansoni*	77%	41.91%	XP_018645017.1	82%	48.33%	XP_018648322.1	55%	46.28%	XP_018650394.1
*Leishmania donovani*	97%	29.61%	XP_003863105.1	44%	35.51%	AYU82972.1	54%	31.85%	XP_003861970.1
*Eimeria acervulina*	58%	34.33%	XP_013246507.1	26%	41.18%	XP_013250312.1	55%	38.83%	XP_013253187
*Toxoplasma gondii*	98%	30.14%	KYK69963.1	33%	37.46%	KFG53298.1	29%	38.92%	KFG34937.1
*Trichinella spiralis*	96%	44.65%	XP_003375066.1	94%	48.33%	KRY40700.1	46%	42.19%	XP_003380019.1
*Nosema bombycis*	ABSENT			29%	35.24%	EOB14601.1	ABSENT		
*Cryptosporidium parvum*	99%	31.78%	QOY43601.1	37%	38.79%	XP_627060.1	54%	30.88%	QOY42883
*Trypanosoma cruzi*	97%	33.87%	KAF8294020.1	32%	38.50%	PWU84353.1	54%	34.55%	EKJ03440.1
Parasite	USP28				CYLD			A20	
	Query coverage	% Identity	Accession ID	Query coverage	% Identity	Accession ID	Query coverage	% Identity	Accession ID
*Plasmodium falciparum*	8%	31.82%	KOB64092.1	ABSENT			ABSENT		
*Schistosoma mansoni*	31%	25.26%	XP_018648322.1	ABSENT			27%	30.63%	XP_018651129.1
*Leishmania donovani*	27%	25.98%	AYU82972.1	ABSENT			ABSENT		
*Eimeria acervulina*	4%	35.85%	XP_013250312.1	ABSENT			ABSENT		
*Toxoplasma gondii*	4%	39.62%	KGF53298.1	ABSENT			ABSENT		
*Trichinella spiralis*	29%	27.20%	KRY40700.1	41%	37.22%	KRY38886.1	34%	33.86%	KRY33887.1
*Nosema bombycis*	31%	25.37%		EOB14601.1	ABSENT		ABSENT		
*Cryptosporidium parvum*	ABSENT				ABSENT		ABSENT		
*Trypanosoma cruzi*	21%		29.20%	EKF30393.1	ABSENT		ABSENT		
Parasite	UCHL3			ATAXIN3			USP2a		
	Query coverage	% Identity	Accession ID	Query coverage	% Identity	Accession ID	Query coverage	% Identity	Accession ID
*Plasmodium falciparum*	96%	33.33%	ETW54267.1	46%	32.94%	ETW17560.1	53%	35.83%	ETW50834.1
*Schistosoma mansoni*	77%	43.09%	XP_018651143.1	78%	41.72%	XP_018650214.1	55%	46.28%	XP_018650394.1
*Leishmania donovani*	96%	36.24%	XP_003861350.1	ABSENT			54%	31.85%	XP_003861970.1
*Eimeria acervulina*	65%	27.71%	XP_013251940.1	ABSENT			55%	38.83%	XP_013253187
*Toxoplasma gondii*	100%	36.36%	XP_002365447.1	55%	35.52%	KHF17592.1	29%	38.92%	KFG34937.1
*Trichinella spiralis*	96%	26.69%	KRY33554.1	80%	38.39%	XP_003374686.1	46%	42.19%	XP_003380019.1
*Nosema bombycis*	ABSENT			ABSENT			ABSENT		
*Cryptosporidium parvum*	90%	28.44%	XP_627216.1	61%	31.74%	XP_627894.1	54%	30.88%	QOY42883
*Trypanosoma cruzi*	97%	39.39%	XP_816838.1	ABSENT			54%	34.55%	EKJ03440.1

HsUSP7 was found to have a higher degree of similarity to the *T. spiralis* protein KRY40700.1 (94% query cover and 48.33% identity) although it is not annotated as a DUB. Following query coverage of 82%, the *S. mansoni* protein XP 018648322.1 was found to share 48.33% identity with HsUSP7. Following query coverage of less than 40%, the rest of the proteins were determined to have a lower identity, ranging from 30% to 40%. The co-crystal structure of human USP7 with small molecule inhibitors has promised allosteric USP7-selective inhibitors with anticancer activity having IC_50_ of <10 nM (Gavory et al., 2018). These small inhibitors can simply be tested as antiparasitic agents against those parasites where similar USP7 domains were conserved. The HsUSP2a protein, with query coverage of 50%, was discovered to share sequence identity ranges between 30 to 45%among all parasites In *N. bombycis*, the HsUSP2a homolog is absent.

Similarly, HsUSP28 protein was found to share sequence identity ranging from 25% to 35% (query coverage of less than 30%) against all parasites. The low query coverage of parasites may attract these DUBs as novel drug targets since their sequence and domains are significantly different from their human counterparts. The HsUSP28 homologue was absent in *C. parvum*. The human cylindromatosis (CYLD) DUB domain, an important regulator of NF-κB, was found to share a significant identity with *Trichinella* protein. Further A20 protein of human macrophages were found to share identity with *T. spiralis* and *S. mansoni* (33.86% and 30.63% identity, respectively) where query coverage was 34% and 27% respectively for these unidentified proteins of the parasite. The human CYLD and A20 homologues were absent in the rest of the parasites. In all parasites except *N. bombycis*, the HsUCHL3 shared 30 to 40% identity with respect to 90% to 100% query coverage. In parasites such as *Plasmodium*, *Schistosoma*, *Toxoplasma*, *Trichinella*, and *Cryptosporidium*, the HsATAXIN3 was shown to share 30 to 40% identity, whereas query coverage was 50 to 80%. In *Leishmania*, *Eimeria*, *Nosema*, and *Trypanosoma* species, the human ATAXIN3 homolog was not found.

We identified that the active sites of some of the parasite DUB proteins are structurally similar to human DUBs proteins, implying that they may play a similar role and be critical for parasite survival based on their DUB activity with conserved catalytic sites. Designed inhibitors against other parasitic DUBs, which are structurally distinct from human DUBs, could also be potential therapeutic targets, especially in the host-parasite infection model, where these inhibitors are expected to kill the parasites only.

## Conclusion and future perspective

The DUB family comprises >100 members subdivided into six classes. The USFDA approval and clinical success of the 26S proteasome inhibitor Velcade (bortezomib) and its successors have established the UPS as a viable target for anti-cancer delivery. However, targeting or inhibition studies of DUBs are much simpler than UPS since deubiquitination is a one-step, ATP-independent process where the enzyme activity (either from cellular extract or from recombinant purified version) can be easily determined using fluorescent conjugated Ub-substrates of Lysine. Even though a simple assay system was available for DUBs and the difference between the parasite and human proteasome encourages further studies, there was surprisingly no detailed study to date about the parasite DUBs and their possible intervention as antiparasitic drug targets. Lack of research and funding for parasitic diseases of which some of which come under NTDs (like leishmaniasis, Chagas disease, etc.) is probably one of the reasons for that. Some isolated studies in *P. falciparum* (Artavanis-Tsakonas et al., 2006), *L. Mexicana* (Sundar and Chakravarty, 2018), *N. bombycis* (Wang et al., 2015), and *C. parvum* (Ju et al., 2014) have indicated the role of DUBs as potential antiparasitic agents. However, to date, there is no DUB inhibitor for parasites which is successfully validated although 20S proteasomal inhibitors for *Plasmodium* and *Leishmania* with co-crystal structures were already developed (Li et al., 2016; Nagle et al., 2020). Further, molecular genetic studies of parasites with knock-out of DUBs and detailed functional studies of native/recombinant DUBs are virtually unexplored so far. Based on our simple *in silico* studies, we have identified a series of putative DUBs across different parasites with variable degrees of conservation for different classes of DUB-containing domains (USP7, USP14, UCH, OTU1/2, etc.) of human. The crystal structure of USP7, USP14, and OTU-domain of Human DUBs with their respective substrates/inhibitors have already given hope that this information can be used for antiparasitic drug targets since similar DUB domains are present in parasites. In humans, UPS and the related DUBs are controlling the quality of proteins which are part of almost all cellular/metabolic processes. The parasites, having digenetic life cycles, may provide more complexity and, therefore, the possibility of developing multi-stage drugs/inhibitors against these DUBs since the expression level and function of these parasitic DUBs may vary drastically based on their life cycle.

## Author contributions

PrK, DM, and RV conceptualized the idea of the manuscript. PrK, PaK, and DM collected information. PrK, DM, and RV written and edited the manuscript. The final manuscript was reviewed and approved for submission by all authors. All authors contributed to the article and approved the submitted version.

## Funding

This research did not receive any specific grant from funding agencies in the public, commercial or not-for-profit sectors. However, Post-doctoral fellowship of PrK and PhD fellowship of PaK is funded by The ministry of chemicals and fertilizers, Department of Pharmaceuticals, Government of India.

## Conflict of interest

The authors declare that the research was conducted in the absence of any commercial or financial relationships that could be construed as a potential conflict of interest.

## Publisher’s note

All claims expressed in this article are solely those of the authors and do not necessarily represent those of their affiliated organizations, or those of the publisher, the editors and the reviewers. Any product that may be evaluated in this article, or claim that may be made by its manufacturer, is not guaranteed or endorsed by the publisher.

## References

[B1] Aleo E. Henderson C. J. Fontanini A. Solazzo B. Brancolini C.. (2006). Identification of new compounds that trigger apoptosome-independent caspase activation and apoptosis. Cancer Res. 66 (18), 9235–9244. doi: 10.1158/0008-5472.CAN-06-0702 16982768

[B2] Alksne L. E. (2002). Virulence as a target for antimicrobial chemotherapy. Expert Opin. Investig. Drugs 11 (8), 1149–1159. doi: 10.1517/13543784.11.8.1149 12150708

[B3] Allen P. C. Fetterer R. H. (2002). Recent advances in biology and immunobiology of eimeria species and in diagnosis and control of infection with these coccidian parasites of poultry. Clin Microbiol. Rev. 15 (1), 58–65. doi: 0.1128/CMR.15.1.58-65.2002 11781266 10.1128/CMR.15.1.58-65.2002 PMC118059

[B4] Al-Shami A. Jhaver K. G. Vogel P. Wilkins C. Humphries J. Davis J. J.. (2010). Regulators of the proteasome pathway, Uch37 and Rpn13, play distinct roles in mouse development. PloS One 5 (10), e13654. doi: 10.1371/journal.pone.0013654. 21048919 PMC2965108

[B5] Altun M. Kramer H. B. Willems L. I. McDermott J. L. Leach C. A. Goldenberg S. J.. (2011). Activity-based chemical proteomics accelerates inhibitor development for deubiquitylating enzymes. Chem Biol. 18 (11), 1401–1412. doi: 10.1016/j.chembiol.2011.08.018 22118674

[B6] Altun M. Walter T. S. Kramer H. B. Herr P. Iphöfer A. Boström J.. (2015). The human otubain2-ubiquitin structure provides insights into the cleavage specificity of poly-ubiquitin-linkages. PloS One 10 (1), e0115344. doi: 10.1371/journal.pone.0115344 25590432 PMC4295869

[B7] Alvar J. Vélez I. D. Bern C. Herrero M. Desjeux P. Cano J.. (2012). Leishmaniasis worldwide and global estimates of its incidence. PloS One 7 (5), e35671. doi: 10.1371/journal.pone.0035671 22693548 PMC3365071

[B8] Ambroggio X. I. Rees D. C. Deshaies R. J. Ploegh H. L.. (2004). JAMM: a metalloprotease-like zinc site in the proteasome and signalosome. Plos Biol. 2 (1). e2. doi: 10.1371/journal.pbio.0020002 14737182 PMC300881

[B9] Amerik A. Y. Hochstrasser M. Hochstrasser M. (2004). Mechanism and function of deubiquitinating enzymes. Biochimica et Biophysica Acta (BBA)-Mol. Cell Res 1695 (1-3), 189–207. doi: 10.1016/j.bbamcr.2004.10.003 15571815

[B10] Artavanis-Tsakonas K. Misaghi S. Comeaux C. A. Catic A. Spooner E. Duraisingh M. T.. (2006). Identification by functional proteomics of a deubiquitinating/deNeddylating enzyme in plasmodium falciparum. Molecular Microbiol. 61 (5), 1187–1195. doi: 10.1111/j.1365-2958.2006.05307.x PMC7168409 16925553

[B11] Atanassov B. S. Koutelou E. Dent S. Y. (2011). The role of deubiquitinating enzymes in chromatin regulation. FEBS Lett. 585 (13), 2016–2023. doi: 10.1016/j.febslet.2010.10.042 20974139 PMC3036772

[B12] Azevedo C. S. Guido B. C. Pereira J. L. Nolasco D. O. Corrêa R. Magalhães K. G.. (2017). Revealing a novel otubain-like enzyme from leishmania infantum with deubiquitinating activity toward K48-linked substrate. Front. Chem. 5, 13. doi: 10.3389/fchem.2017.00013. 28386537 PMC5362604

[B13] Barban do Patrocínio A.. (2021). Deubiquitinating enzymes as possible drug targets for schistosomiasis. Acta Trop. 217, 105856. 33577811 10.1016/j.actatropica.2021.105856

[B14] Bhattacharjee M. Adhikari N. Sudhakar R. Rizvi Z. Das D. Palanimurugan R.. (2020). Characterization of plasmodium falciparum NEDD8 and identification of cullins as its substrates. Scientific Rep. 10 (1), 1–18. doi: 10.1038/s41598-020-77001-5. PMC7677368 33214620

[B15] Bishop P. Rocca D. Henley J.M.J.B.J. (2016). Ubiquitin c-terminal hydrolase L1 (UCH-L1): structure, distribution and roles in brain function and dysfunction. Biochem. J. 473 (16), 2453–2462. doi: 10.1042/BCJ20160082 27515257 PMC4980807

[B16] Burnett B. Li F. Pittman R. N. (2003). The polyglutamine neurodegenerative protein ataxin-3 binds polyubiquitylated proteins and has ubiquitin protease activity. Hum Mol. Gene 12 (23), 3195–3205. doi: 0.1093/hmg/ddg344 10.1093/hmg/ddg344 14559776

[B17] Chen X. Walters K. J. (2015). Structural plasticity allows UCH37 to be primed by RPN13 or locked down by INO80G. Mol. Cell. 57 (5), 767–768. doi: 10.1016/j.molcel.2015.02.025 25747657 PMC6296220

[B18] Clague M. J. Urbé S. Komander D. (2019). Breaking the chains: deubiquitylating enzyme specificity begets function. Nat Rev Mol. Cell Biol. 20 (6), 338–352. doi: 10.1038/s41580-019-0099-1 30733604

[B19] Clark E. L. Macdonald S.E. Thenmozhi V. Kundu K. Garg R. Kumar S. (2016). Cryptic eimeria genotypes are common across the southern but not northern hemisphere. Int. J. Parasitol. 46 (9), 537–544. doi: 10.1016/j.ijpara.2016.05.006 27368611 PMC4978698

[B20] Colland F. (2010). The therapeutic potential of deubiquitinating enzyme inhibitors. Biochem Soc Transactions. 38 (1), 137–143. doi: 10.1042/BST0380137 20074048

[B21] D'Arcy P. Wang X. Linder S.. (2015). Deubiquitinase inhibition as a cancer therapeutic strategy. Pharmacol. Therapeut. 147, 32–54. doi: 10.1016/j.pharmthera.2014.11.002 25444757

[B22] Damianou A. Burge R. J. Catta-Preta C. M. Geoghegan V. Nievas Y. R. Newling K.. (2020). Essential roles for deubiquitination in leishmania life cycle progression. PLoS Pathog. 16 (6. doi: 10.1371/journal.ppat.1008455 PMC7319358 32544189

[B23] Daviet L. Colland F. J. B. (2008). Targeting ubiquitin specific proteases for drug discovery. Biochimie 90 (2), 270–283. doi: 10.1016/j.biochi.2007.09.013 17961905

[B24] De Monerri N. C. S. Yakubu R. R. Chen A. L. Bradley P. J. Nieves E. Weiss L. M.. (2015). The ubiquitin proteome of toxoplasma gondii reveals roles for protein ubiquitination in cell-cycle transitions. Cell Host Microbe. 18 (5), 621–633. doi: 10.1016/j.chom.2015.10.014 26567513 PMC4968887

[B25] Deng L. Meng T. Chen L. Wei W. Wang P.. (2020). The role of ubiquitination in tumorigenesis and targeted drug discovery. Signal Transduction and Targeted Ther. 5 (1), 1–28. doi: 10.1038/s41392-020-0107-0 PMC7048745 32296023

[B26] Deol K. K. Lorenz S. Strieter E. R. (2019). Enzymatic logic of ubiquitin chain assembly. Front. Physiol. 10, 835. doi: 10.3389/fphys.2019.00835 31333493 PMC6624479

[B27] Dhara A. de Paula Baptista R. Kissinger J. C. Snow E. C. Sinai A. P.. (2017). Ablation of an ovarian tumor family deubiquitinase exposes the underlying regulation governing the plasticity of cell cycle progression in toxoplasma gondii. MBio 8 (6), e01846-17. doi: 10.1128/mBio.01846-17 29162714 PMC5698556

[B28] Didier E. S. Snowden K. F. Shadduck J. A. (1998). Biology of microsporidian species infecting mammals. Adv. Parasitol. 40, 283–320. doi: 10.1016/s0065-308x(08)60125-6 9554077

[B29] do Carmo Costa M. Paulson H. L. (2012). Toward understanding machado–Joseph disease. Prog. neurobiol. 97 (2), 239–257. doi: 10.1016/j.pneurobio.2011.11.006 22133674 PMC3306771

[B30] Dong Y. Zhang S. Wu Z. Li X. Wang W. L. Zhu Y.. (2019). Cryo-EM structures and dynamics of substrate-engaged human 26S proteasome. Nature 565 (7737), 49–55. doi: 10.1038/s41586-018-0736-4 30479383 PMC6370054

[B31] do Patrocinio A. B. Cabral F. J. Bitencourt A. L. Brigato O. M. Magalhães L. G. de Lima Paula L. A.. (2020). Inhibition of 19S proteasome deubiquitinating activity in schistosoma mansoni affects viability, oviposition, and structural changes. Parasitol. Res. 119 (7), 2159–2176. doi: 10.1007/s00436-020-06686-4 32424554

[B32] Dubey J. Jones J. L. (2008). Toxoplasma gondii infection in humans and animals in the united states. Int. jour. parasito. 38 (11), 1257–1278. doi: 10.1016/j.ijpara.2008.03.007 18508057

[B33] Duong B. H. Onizawa M. Oses-Prieto J. A Advincula R. Burlingame A. Malynn B. A.. (2015). A20 restricts ubiquitination of pro-interleukin-1β protein complexes and suppresses NLRP3 inflammasome activity. Immunity 42 (1), 55–67. doi: 10.1016/j.immuni.2014.12.031 25607459 PMC4302274

[B34] Ebner P. Versteeg G. A. Ikeda F.. (2017). Ubiquitin enzymes in the regulation of immune responses. Crit. Rev. Biochem. Mol. Biol. 52 (4), 425–460. doi: 10.1080/10409238.2017.1325829 28524749 PMC5490640

[B35] Edelmann M. J. Iphöfer A. Akutsu M. Altun M. Di Gleria K. Kramer H. B.. (2009). Structural basis and specificity of human otubain 1-mediated deubiquitination. Biochem. 418 (2), 379–390. doi: 10.1042/BJ20081318 18954305

[B36] Etlinger J. D. Goldberg A. L. (1977). A soluble ATP-dependent proteolytic system responsible for the degradation of abnormal proteins in reticulocytes. PNAS 74 (1), 54–58. doi: 10.1073/pnas.74.1.54 264694 PMC393195

[B37] Fabre M. Beiting D. P. Bliss S. K. Appleton J. A.. (2009). Immunity to trichinella spiralis muscle infection. Vet. Parasitol. 159 (3-4), 245–248. doi: 10.1016/j.vetpar.2008.10.051 19070961 PMC3449155

[B38] Farshi P. Deshmukh R. R. Nwankwo J. O. Arkwright R. T. Cvek B. Liu J.. (2015). Deubiquitinases (DUBs) and DUB inhibitors: a patent review. Expert Opin Ther. Pat. 25 (10), 1191–1208. doi: 10.1517/13543776.2015.1056737 26077642 PMC4834700

[B39] Fiil B. K. Damgaard R. B. Wagner S. A. Keusekotten K. Fritsch M. Bekker-Jensen S.. (2013). OTULIN restricts Met1-linked ubiquitination to control innate immune signaling. Mol. Cell. 50 (6), 818–830. doi: 10.1016/j.molcel.2013.06.004 23806334 PMC4194427

[B40] Fishelson Z. Amiri P. Friend D. S. Marikovsky M. Petitt M. Newport G.. (1992). Schistosoma mansoni: cell-specific expression and secretion of a serine protease during development of cercariae. Exp. Parasitol. 75 (1), 87–98. doi: 10.1016/0014-4894(92)90124-s 1639166

[B41] Frickel E. M. Quesada V. Muething L. Gubbels M. J. Spooner E. Ploegh H.. (2007). Apicomplexan UCHL3 retains dual specificity for ubiquitin and Nedd8 throughout evolution. Cell Microbiol. 9 (6), 1601–1610. doi: 10.1111/j.1462-5822.2007.00896.x 17371404

[B42] Gavory G. O'Dowd C. R. Helm M. D. Flasz J. Arkoudis E. Dossang A.. (2018). Discovery and characterization of highly potent and selective allosteric USP7 inhibitors. Nat. Chem. Biol. 14 (2), 118–125. doi: 10.1038/nchembio.2528 29200206

[B43] Ghosh S. Farr L. Singh A. Leaton L. A. Padalia J. Shirley D. A.. (2020). COP9 signalosome is an essential and druggable parasite target that regulates protein degradation. PLoS Pathog. 16 (9. doi: 10.1371/journal.ppat.1008952 PMC7531848 32960936

[B44] Goldknopf I. L. Busch H. (1977). Isopeptide linkage between nonhistone and histone 2A polypeptides of chromosomal conjugate-protein A24. Natl. Acad. Sci. U. S. A. 74 (3), 864–868. doi: 10.1073/pnas.74.3.864 PMC430507 265581

[B45] Gualdrón-López M. Chevalier N. Van Der Smissen P. Courtoy P. J. Rigden D. J. Michels P. A.. (2013). Ubiquitination of the glycosomal matrix protein receptor PEX5 in trypanosoma brucei by PEX4 displays novel features. Biochim. Biophys. Acta. 1833 (12), 3076–3092. doi: 10.1016/j.bbamcr.2013.08.008 23994617

[B46] Gupta A. K. Ghosh K. Palit S. Barua J. Das P. K. Ukil A.. (2017). Leishmania donovani inhibits inflammasome-dependent macrophage activation by exploiting the negative regulatory proteins A20 and UCP2. FASEB J. 31 (11), 5087–5101. doi: 0.1096/fj.201700407R 28765172 10.1096/fj.201700407R

[B47] Gupta I. Aggarwal S. Singh K. Yadav A. Khan S.. (2018). Ubiquitin proteasome pathway proteins as potential drug targets in parasite trypanosoma cruzi. Sci. Rep. 8 (1), 1–12. doi: 10.1038/s41598-018-26532-z 29849031 PMC5976635

[B48] Hermanns T. Pichlo C. Woiwode I. Klopffleisch K. Witting K. F. Ovaa H.. (2018). A family of unconventional deubiquitinases with modular chain specificity determinants. Nat. Commun. 9 (1), 1–13. doi: 10.1038/s41467-018-03148-5 29476094 PMC5824887

[B49] Hill D. E. Chirukandoth S. Dubey J. P. (2005). Biology and epidemiology of toxoplasma gondii in man and animals. Anim Health Res. Rev. 6 (1), 41–61. doi: 10.1079/ahr2005100 16164008

[B50] Huang X. Dixit V. M. (2016). Drugging the undruggables: exploring the ubiquitin system for drug development. Cell Res. 26 (4), 484–498. doi: 10.1038/cr.2016.31 27002218 PMC4822129

[B51] Hu M. Li P. Li M. Li W. Yao T. Wu J. W.. (2002). Crystal structure of a UBP-family deubiquitinating enzyme in isolation and in complex with ubiquitin aldehyde. Cell 111 (7), 1041–1054. doi: 10.1016/s0092-8674(02)01199-6 12507430

[B52] Hunt P. Afonso A. Creasey A. Culleton R. Sidhu A. B. Logan J.. (2007). Gene encoding a deubiquitinating enzyme is mutated in artesunate-and chloroquine-resistant rodent malaria parasites. Cell 65 (1), 27–40. doi: 10.1111/j.1365-2958.2007.05753.x PMC1974797 17581118

[B53] Ikeda F. Dikic I. (2008). Atypical ubiquitin chains: new molecular signals. EMBO Rep. 9 (6), 536–542.doi: 10.1038/embor.2008.93 18516089 PMC2427391

[B54] Ju H.-L. Kang J. M. Noh H. S. Kim D. R. Hong Y. Sohn W. M. (2014). Characterization of a novel otubain-like cysteine protease of cryptosporidium parvum. Parasitol. Int. 63 (4), 580–583. doi: 10.1016/j.parint.2014.03.005 24709083

[B55] Kane R. C. Bross P. F. Farrell A.T. Pazdur R.. (2003). Velcade®: US FDA approval for the treatment of multiple myeloma progressing on prior therapy. The oncologist. 8 (6), 508–513. 14657528 10.1634/theoncologist.8-6-508

[B56] Karpiyevich M. Adjalley S Mol M Ascher D. B. Mason B. van der Heden van Noort G. J.. (2019). Nedd8 hydrolysis by UCH proteases in plasmodium parasites. PLoS Pathog. 15 (10. doi: 10.1371/journal.ppat.1008086 PMC6837540 31658303

[B57] Kim M. Otsubo R. Morikawa H. Nishide A. Takagi K. Sasakawa C.. (2014). Bacterial effectors and their functions in the ubiquitin-proteasome system: insight from the modes of substrate recognition. Cell 3 (3), 848–864. doi: 10.3390/cells3030848 PMC4197628 25257025

[B58] Kim R. Q. van Dijk W. J. Sixma T. (2016). Structure of USP7 catalytic domain and three ubl-domains reveals a connector α-helix with regulatory role. J. Struct. Biol. 195 (1), 11–18. doi: 10.1016/j.jsb.2016.05.005. 27183903

[B59] Komander D. Clague M. J. Sylvie Urbé M. J. C. (2009). Breaking the chains: structure and function of the deubiquitinases. Nat. Rev. Mol. Cell Biol. 10 (8), 550–563. doi: 10.1038/nrm2731 19626045

[B60] Komander D. Rape M. (2012). The ubiquitin code. Annu. Rev. Biochem. 81, 203–229. doi: 10.1146/annurev-biochem-060310-170328 22524316

[B61] Kors S. Geijtenbeek K. Reits E. Schipper-Krom S. (2019). Regulation of proteasome activity by (post-) transcriptional mechanisms. Front. Mol. Biosci. 16 (6), 48. doi: 10.3389/fmolb.2019.00048 PMC6646590 31380390

[B62] Kristariyanto Y. A. Abdul Rehman S. A. Weidlich S. Knebel A. Kulathu Y.. (2017). A single MIU motif of MINDY-1 recognizes K48-linked polyubiquitin chains. EMBO Rep. 18 (3), 392–402. doi: 10.15252/embr.201643205 28082312 PMC5331195

[B63] Kubes P. Jenne C. (2018). Immune responses in the liver. Annu. Rev. Immunol. 36, 247–277. doi: 10.1146/annurev-immunol-051116-052415 29328785

[B64] Kusotera T. Nhengu T. G. (2020). Coronavirus-19 malaria: great mimics. Afr. J. Prim. Health Care Fam. Med. 12 (1), 1–3. doi: 10.4102/phcfm.v12i1.2501 PMC7479414 32787398

[B65] Lee J.-K. Chang N. Yoon Y. Yang H. Cho H. Kim E.. (2015). USP1 targeting impedes GBM growth by inhibiting stem cell maintenance and radioresistance. Neuro. Oncol. 18 (1), 37–47. doi: 10.1093/neuonc/nov091 26032834 PMC4677407

[B66] Li H. O’Donoghue A. J. van der Linden W.A. Xie S. C. Yoo E. Foe I. T.. (2016). Structure-and function-based design of plasmodium-selective proteasome inhibitors. Nature. 530 (7589), 233–236. doi: 10.1038/nature16936 26863983 PMC4755332

[B67] Li Q. Ye C. Tian T. Jiang Q. Zhao P. Wang X.. (2022). The emerging role of ubiquitin-specific protease 20 in tumorigenesis and cancer therapeutics. Cell Death Dis. 13 (5), 1–11. doi: 10.1038/s41419-022-04853-2 PMC9068925 35508480

[B68] Liu D. Song Z. Wang X. Ouyang L.. (2020). Ubiquitin c-terminal hydrolase L5 (UCHL5) accelerates the growth of endometrial cancer *via* activating the wnt/β-catenin signaling pathway. Front. Oncol. 10, 865. doi: 10.3389/fonc.2020.00865 32596150 PMC7300206

[B69] Liwocha J. Krist D. T. van der Heden van Noort G. Hansen F. M. Truong V. H. Karayel O.. (2021). Linkage-specific ubiquitin chain formation depends on a lysine hydrocarbon ruler. Nat. Chem. Biol. 17 (3), 272–279. doi: 10.1038/s41589-020-00696-0 33288957 PMC7904580

[B70] Lou S.-C. Wetzel S. Zhang H. Crone E. W. Lee Y. T. Jackson S. E.. (2016). The knotted protein UCH-L1 exhibits partially unfolded forms under native conditions that share common structural features with its kinetic folding intermediates. J. Mol. Biol. 428 (11), 2507–2520. doi: 10.1016/j.jmb.2016.04.002 27067109

[B71] Luo K. Li L. Li Y. Wu C. Yin Y. Chen Y.. (2016). A phosphorylation–deubiquitination cascade regulates the BRCA2–RAD51 axis in homologous recombination. Genes Dev. 30 (23), 2581–2595. doi: 10.1101/gad.289439 27941124 PMC5204351

[B72] Ma X. Liu B. Gong Z. Qu Z. Cai J.. (2021). Phosphoproteomic comparison of four eimeria tenella life cycle stages. Int. J. Mol. Sci. 22 (22), 12110. doi: 10.3390/ijms222212110 34829991 PMC8624187

[B73] Maiti T. K. Permaul M. Boudreaux D. A. Mahanic C. Mauney S. Das C.. (2011). Crystal structure of the catalytic domain of UCHL5, a proteasome-associated human deubiquitinating enzyme, reveals an unproductive form of the enzyme. FEBS J. 278 (24), 4917–4926. doi: 0.1111/j.1742-4658.2011.08393.x 21995438 10.1111/j.1742-4658.2011.08393.x PMC3336103

[B74] Majumdar I. Paul J. J. A. (2014). The deubiquitinase A20 in immunopathology of autoimmune diseases. Autoimmunity. 47 (5), 307–319. doi: 10.3109/08916934.2014.900756 24673262

[B75] Makarova K. S. Aravind L. Koonin E. V. (2000). A novel superfamily of predicted cysteine proteases from eukaryotes, viruses and chlamydia pneumoniae. Trends Biochem. Sci. 25 (2), 50–52. doi: 0.1016/s0968-0004(99)01530-3 10664582 10.1016/s0968-0004(99)01530-3

[B76] Menard D. Dondorp A. (2017). Antimalarial drug resistance: a threat to malaria elimination. Cold Spring Harb. Perspect. Med. 7 (7), a025619. doi: 10.1101/cshperspect.a025619 28289248 PMC5495053

[B77] Messick T. E. Russell N. S. Iwata A. J. Sarachan K. L Shiekhattar R. Shanks J. R.. (2008). Structural basis for ubiquitin recognition by the Otu1 ovarian tumor domain protein. J. Biol. Chem. 283 (16), 11038–11049. doi: 10.1074/jbc.M704398200 18270205 PMC2447653

[B78] Mitreva M. Jasmer D. P. (2010). Trichinella spiralis: genomic application to control a zoonotic nematode. Infect. Disord. Drug Targets 10 (5), 376–384. doi: 10.2174/187152610793180830 20701572

[B79] Miyake Y. Tanaka K. Fukushima W. Kiyohara C. Sasaki S. Tsuboi Y.. (2012). UCHL1 S18Y variant is a risk factor for parkinson’s disease in Japan. BMC Neurol. 12 (1), 1–8. doi: 10.1186/1471-2377-12-62 22839974 PMC3488468

[B80] Mondal M. Conole D. Nautiyal J. Tate E. W.. (2022). UCHL1 as a novel target in breast cancer: emerging insights from cell and chemical biology. Br. J. Cancer. 126 (1), 24–33. doi: 10.1038/s41416-021-01516-5 34497382 PMC8727673

[B81] Mulas F. Wang X. Song S. Nishanth G. Yi W. Brunn A.. (2021). The deubiquitinase OTUB1 augments NF-κB-dependent immune responses in dendritic cells in infection and inflammation by stabilizing UBC13. Cell. Mol. Immunol. 18 (6), 1512–1527. doi: 10.1038/s41423-020-0362-6 32024978 PMC8167118

[B82] Nagle A. Biggart A. Be C. Srinivas H. Hein A. Caridha D.. (2020). Discovery and characterization of clinical candidate LXE408 as a kinetoplastid-selective proteasome inhibitor for the treatment of leishmaniases. J. Med. Chem. 63 (19), 10773–10781. doi: 10.1021/acs.jmedchem.0c00499 32667203 PMC7549094

[B83] Nanao M. H. Tcherniuk S. O. Chroboczek J. Dideberg O. Dessen A. Balakirev M. Y.. (2004). Crystal structure of human otubain 2. EMBO Rep. 5 (8), 783–788. doi: 10.1038/sj.embor.7400201 15258613 PMC1299112

[B84] Ndao M. Nath-Chowdhury M. Sajid M. Marcus V. Mashiyama S. T. Sakanari J.. (2013). A cysteine protease inhibitor rescues mice from a lethal cryptosporidium parvum infection. Antimicrob. Agents Chemother. 57 (12), 6063–6073. doi: 10.1128/AAC.00734-13 24060869 PMC3837922

[B85] Ndubaku C. Tsui V. (2015). Inhibiting the deubiquitinating enzymes (DUBs) miniperspective. J. Med. Chem. 58 (4), 1581–1595. doi: 10.1021/jm501061a 25364867

[B86] Nicholson B. Kumar S. Agarwal S. Eddins M. J. Marblestone J. G. Wu J.. (2014). Discovery of therapeutic deubiquitylase effector molecules: current perspectives. J. Biomol. Screen. 19 (7), 989–999. doi: 10.1177/1087057114527312 24632661

[B87] Pereira R. V. de Souza Gomes M. Costa M. P. Jannotti Passos L. K. de Castro Borges W. Guerra-Sá R.. (2015a). MJD and OTU deubiquitinating enzymes in schistosoma mansoni. Parasitol. Res. 114 (8), 2835–2843. doi: 10.1007/s00436-015-4484-1 25924794

[B88] Pereira R. V. de Gomes S. M. Olmo R. P. Souza D. M. Cabral F. J. Jannotti-Passos L. K.. (2015b). Ubiquitin-specific proteases are differentially expressed throughout the schistosoma mansoni life cycle. Parasit. Vectors 8, 349. doi: 10.1186/s13071-015-0957-4 26112833 PMC4485857

[B89] Pickart C. M. Cohen R. E. (2004). Proteasomes and their kin: proteases in the machine age. Nat. Rev. Mol. Cell Biol. 5 (3), 177–187. doi: 10.1038/nrm1336 14990998

[B90] Ponder E. L. Bogyo M. (2007). Ubiquitin-like modifiers and their deconjugating enzymes in medically important parasitic protozoa. Eukaryot Cell. 6 (11), 1943–1952. doi: 10.1128/EC.00282-07 17905920 PMC2168404

[B91] Ponts N. Yang J. Chung D. W. Prudhomme J. Girke T. Horrocks P.. (2008). Deciphering the ubiquitin-mediated pathway in apicomplexan parasites: a potential strategy to interfere with parasite virulence. PLos. One 3 (6. doi: 10.1371/journal.pone.0002386 PMC2408969 18545708

[B92] Pozhidaeva A. Bezsonova I. (2019). USP7: Structure, substrate specificity, and inhibition. DNA Repair (Amst). 76, 30–39. doi: 0.1016/j.dnarep.2019.02.005 30807924 10.1016/j.dnarep.2019.02.005 PMC6481172

[B93] Rabut G. Peter M. (2008). Function and regulation of protein neddylation. EMBO Rep. 9 (10), 969–976. doi: 10.1038/embor.2008.183 18802447 PMC2572130

[B94] Ragland M. Hutter C Zabetian C Edwards K.. (2009). Association between the ubiquitin carboxyl-terminal esterase L1 gene (UCHL1) S18Y variant and parkinson's disease: a HuGE review and meta-analysis. Am. J. Epidemiol. 170 (11), 1344–1357. doi: 10.1093/aje/kwp288 19864305 PMC2778765

[B95] Ramanathan H. N. Ye Y. (2012). Cellular strategies for making monoubiquitin signals. Crit. Rev. Biochem. Mol. Biol. 47 (1), 17–28. doi: 10.3109/10409238.2011.620943 21981143 PMC3476054

[B96] Rehman S. A. Kristariyanto Y. A. Choi S. Y. Nkosi P. J Weidlich S. Labib K.. (2016). MINDY-1 is a member of an evolutionarily conserved and structurally distinct new family of deubiquitinating enzymes. Mol. Cell. 63 (1), 146–155. doi: 10.1016/j.molcel.2016.05.009 27292798 PMC4942677

[B97] Reyes-Turcu F. E. Ventii K. H. Wilkinson K. D. (2009). Regulation and cellular roles of ubiquitin-specific deubiquitinating enzymes. Annu. Rev. Biochem. 78, 363–397. doi: 0.1146/annurev.biochem.78.082307.091526 19489724 10.1146/annurev.biochem.78.082307.091526 PMC2734102

[B98] Ronau J. A. Beckmann J. F. Hochstrasser M. (2016). Substrate specificity of the ubiquitin and ubl proteases. Cell. Res. 26 (4), 441–456. doi: 10.1038/cr.2016.38 27012468 PMC4822132

[B99] Sadowski M. Suryadinata R. Tan A. R. Roesley S. N. Sarcevic B.. (2012). Protein monoubiquitination and polyubiquitination generate structural diversity to control distinct biological processes. IUBMB Life. 64 (2), 136–142. doi: 10.1002/iub.589 22131221

[B100] Shah S. (2012). Jaypee brothers 12, 321–323.

[B101] Shinji S. Naito Z. Ishiwata S. Ishiwata T. Tanaka N. Furukawa K.. (2006). Ubiquitin-specific protease 14 expression in colorectal cancer is associated with liver and lymph node metastases. Oncol. Rep. 15 (3), 539–543. 16465409

[B102] Sigismund S. Polo S. Di Fiore P. P. (2004). Signaling through monoubiquitination. Curr. Top. Microbiol. Immunol. 149–185. doi: 10.1007/978-3-540-69494-6_6 15645713

[B103] Simwela N. V. Hughes K. R Rennie M. T Barrett M. P Waters A. P.. (2021). Mammalian deubiquitinating enzyme inhibitors display *in vitro* and *in vivo* activity against malaria parasites and potentiate artemisinin action. ACS Infect. Dis. 7 (2), 333–346. doi: 10.1021/acsinfecdis.0c00580 33400499

[B104] Siyah P. Akgol S. Durdagi S. Kocabas F.. Identification of viral OTU-like plasmodium parasite proteases and development of antimalarial DUB inhibitors.2021

[B105] Song Y. Y. Wang L. A. Ren H. N. Qi X. Sun G. G.. (2018). Cloning, expression and characterisation of a cysteine protease from trichinella spiralis 65, 1–11. doi: 10.14411/fp.2018.007 29905572

[B106] Song Z. Li J. Zhang L. Deng J. Fang Z. Xiang X.. (2019). UCHL3 promotes pancreatic cancer progression and chemo-resistance through FOXM1 stabilization. Am. J. Cancer Res 9 (9), 1970. 31598398 PMC6780670

[B107] Song L. Luo Z.-Q. (2019). Post-translational regulation of ubiquitin signaling. J Cell Biol. 218 (6), 1776–1786. doi: 10.1083/jcb.201902074 31000580 PMC6548142

[B108] Stirewalt M. A. (1974). Schistosoma mansoni: cercaria to schistosomule. Adv. Parasitol. Adv. Parasitol. 12, 115–182. doi: 10.1016/s0065-308x(08)60388-7 4141581

[B109] Sun L. Chen Z. J. (2004). The novel functions of ubiquitination in signaling. Curr Opin Cell Biol. 16 (2), 119–126. doi: 10.1016/j.ceb.2004.02.005 15196553

[B110] Sundar S. Chakravarty J. (2018). “Visceral leishmaniasis,” in Drug resistance in leishmania parasites (Springer), 159–176.

[B111] Suresh H. G. Pascoe N. Andrews B. (2020). The structure and function of deubiquitinases: Lessons from budding yeast. Open Biol. 10 (10), 200279. doi: 10.1098/rsob.200279 33081638 PMC7653365

[B112] Wada T. Yamashita Y. Saga Y. Takahashi K. Koinuma K. Choi Y. L.. (2009). Screening for genetic abnormalities involved in ovarian carcinogenesis using retroviral expression libraries. Int. J. Oncol. 35 (5), 973–976. doi: 10.3892/ijo_00000410 19787249

[B113] Wakelin D. (1993). Trichinella spiralis: immunity, ecology, and evolution. J. Parasitol. p, 488–494. 8331470

[B114] Wang Y. Dang X. Luo B. Li C. Long M. Li T.. (2015). Characterization of a novel otubain-like protease with deubiquitination activity from nosema bombycis (Microsporidia). Parasitol. Res. 114 (10), 3759–3766. doi: 10.1007/s00436-015-4624-7 26177898

[B115] Wang P. Li J. Gong P. Wang W. Ai Y. Zhang X.. (2018). An OTU deubiquitinating enzyme in eimeria tenella interacts with eimeria tenella virus RDRP. Parasites vectors. 11 (1), 1–11. doi: 10.1186/s13071-018-2626-x 29386062 PMC5793433

[B116] Wang P. Gong P. Wang W. Li J. Ai Y. Zhang X.. (2019). An eimeria acervulina OTU protease exhibits linkage-specific deubiquitinase activity. Parasitol. Res. 118 (1), 47–55. doi: 10.1007/s00436-018-6113-2 30415394

[B117] Wang R. Smith J. D. Kappe S. H. (2009). Advances and challenges in malaria vaccine development. Expert. Rev. Mol. Med. 11, e39. doi: 10.1017/S1462399409001318 20003658 PMC2943423

[B118] Wei R. Liu X. Yu W. Yang T Cai W. Liu J.. (2015). Deubiquitinases in cancer. Oncotarget 6 (15), 12872. doi: 10.18632/oncotarget.3671 25972356 PMC4536986

[B119] White R. R. Miyata S. Papa E. Spooner E. Gounaris K. Selkirk M. E.. (2011). Characterisation of the trichinella spiralis deubiquitinating enzyme, TsUCH37, an evolutionarily conserved proteasome interaction partner. PLoS Negl. Trop. Dis. 5 (10. doi: 10.1371/journal.pntd.0001340 PMC3186758 22013496

[B120] Wilkinson K. D. (1997). Regulation of ubiquitin-dependent processes by deubiquitinating enzymes. FASEB J. 11 (14), 1245–1256. doi: 10.1096/fasebj.11.14.9409543 9409543

[B121] Williams B. A. P. (2009). Unique physiology of host–parasite interactions in microsporidia infections. Cell Microbiol. 11 (11), 1551–1560. doi: 10.1111/j.1462-5822.2009.01362.x 19673893

[B122] Xu D. Shan B. Lee B. H. Zhu K Zhang T. Sun H.. (2015). Phosphorylation and activation of ubiquitin-specific protease-14 by akt regulates the ubiquitin-proteasome system. Elife. 4. doi: 10.7554/eLife.10510 PMC4733041 26523394

[B123] Zaheer T. Imran M. Abbas R. Z. Zaheer I. Malik M. A.. (2021). Avian cryptosporidiosis and its zoonotic significance in Asia. World's Poultry Science Journal. 77 (1), 55–70. doi: 10.1080/00439339.2020.1866961

